# Ethnicity-specific BMI cutoffs for obesity based on type 2 diabetes risk in England: a population-based cohort study

**DOI:** 10.1016/S2213-8587(21)00088-7

**Published:** 2021-07

**Authors:** Rishi Caleyachetty, Thomas M Barber, Nuredin Ibrahim Mohammed, Francesco P Cappuccio, Rebecca Hardy, Rohini Mathur, Amitava Banerjee, Paramjit Gill

**Affiliations:** a Nuffield Department of Population Health, University of Oxford, Oxford, UK; b Warwick Medical School, University of Warwick, Coventry, UK; c Warwickshire Institute for the Study of Diabetes, Endocrinology and Metabolism, University Hospitals Coventry and Warwickshire NHS Trust, Coventry, UK; d MRC Unit The Gambia at London School of Hygiene and Tropical Medicine, Fajara, The Gambia; e Social Research Institute, University College London, London, UK; f Institute of Health Informatics, University College London, London, UK; g Department of Non-Communicable Disease Epidemiology, London School of Hygiene and Tropical Medicine, London, UK

## Abstract

**Background:**

National and global recommendations for BMI cutoffs to trigger action to prevent obesity-related complications like type 2 diabetes among non-White populations are questionable. We aimed to prospectively identify ethnicity-specific BMI cutoffs for obesity based on the risk of type 2 diabetes that are risk-equivalent to the BMI cutoff for obesity among White populations (≥30 kg/m^2^).

**Methods:**

In this population-based cohort study, we used electronic health records across primary care (Clinical Practice Research Datalink) linked to secondary care records (Hospital Episodes Statistics) from a network of general practitioner practices in England. Eligible participants were aged 18 years or older, without any past or current diagnosis of type 2 diabetes, had a BMI of 15·0–50·0 kg/m^2^ and complete ethnicity data, were registered with a general practitioner practice in England at any point between Sept 1, 1990, and Dec 1, 2018, and had at least 1 year of follow-up data. Patients with type 2 diabetes were identified by use of a CALIBER phenotyping algorithm. Self-reported ethnicity was collapsed into five main categories. Age-adjusted and sex-adjusted negative binomial regression models, with fractional polynomials for BMI, were fitted with incident type 2 diabetes and ethnicity data.

**Findings:**

1 472 819 people were included in our study, of whom 1 333 816 (90·6%) were White, 75 956 (5·2%) were south Asian, 49 349 (3·4%) were Black, 10 934 (0·7%) were Chinese, and 2764 (0·2%) were Arab. After a median follow-up of 6·5 years (IQR 3·2–11·2), 97 823 (6·6%) of 1 472 819 individuals were diagnosed with type 2 diabetes. For the equivalent age-adjusted and sex-adjusted incidence of type 2 diabetes at a BMI of 30·0 kg/m^2^ in White populations, the BMI cutoffs were 23·9 kg/m^2^ (95% CI 23·6–24·0) in south Asian populations, 28·1 kg/m^2^ (28·0–28·4) in Black populations, 26·9 kg/m^2^ (26·7–27·2) in Chinese populations, and 26·6 kg/m^2^ (26·5–27·0) in Arab populations.

**Interpretation:**

Revisions of ethnicity-specific BMI cutoffs are needed to ensure that minority ethnic populations are provided with appropriate clinical surveillance to optimise the prevention, early diagnosis, and timely management of type 2 diabetes.

**Funding:**

National Institute for Health Research.

## Introduction

BMI is an established way of classifying the degree of excess weight in an individual. Nearly three decades ago in 1993, a WHO committee of experts proposed BMI cutoffs of 25·0–29·9 kg/m^2^ for overweight grade 1, 30·0–39·9 kg/m^2^ for overweight grade 2 (now termed obesity class I), and 40·0 kg/m^2^ or more for overweight grade 3 (now termed obesity class III).^1^ The suggested BMI cutoff now used to define obesity (≥30 kg/m^2^) was developed from observational studies in Europe and the USA of exclusively White populations and based on the association between BMI and mortality. Subsequently, there has been increasing evidence of a high prevalence of type 2 diabetes among Asian populations at a lower BMI than in White populations.^2^, ^3^ In response to these emerging data, WHO recommended lowering the BMI cutoffs for defining obesity in south Asian populations to optimise the identification of cardiometabolic risk in this group.^3^ Originating from a WHO expert consultation in 2004,^3^ WHO, and, subsequently, the National Institute for Health and Care Excellence (NICE), recommended a BMI cutoff of 27·5 kg/m^2^ be used for south Asian and Chinese populations to trigger the implementation of lifestyle interventions.^3^, ^4^ The expert consultation recalculated BMI cutoffs based on the measurement of percentage body fat, which is typically higher in Asian people than in White people, from studies done in China, Hong Kong, Indonesia, Japan, Singapore, and Thailand.^3^ Despite the importance of identifying BMI cutoffs for obesity at which adverse outcomes, such as type 2 diabetes, are more likely to occur and producing clinically relevant guidelines for patient care, WHO made recommendations with no or sparse data on the association of BMI with type 2 diabetes and without data on Black, south Asian, and Arab populations.

Type 2 diabetes can be prevented or delayed through dietary change, physical activity, or the use of metformin.^5^ The early use of other antihyperglycaemic therapies reduces the risk of long-term complications from type 2 diabetes via improved glycaemic control.^6^, ^7^, ^8^, ^9^ However, these benefits cannot be fully realised if current WHO and NICE recommendations for obesity under-recognise the risk of developing type 2 diabetes in minority ethnic populations.

**Research in context**

**Evidence before this study**

WHO and the National Institute for Health and Care Excellence (NICE) in England both recommend a BMI cutoff of 27·5 kg/m^2^ to trigger action to reduce the risk of obesity-related conditions, such as type 2 diabetes, in south Asian and Chinese populations. This recommendation is based on a sparse evidence base and therefore might be inappropriate for some minority ethnic groups. Previous studies have attempted to identify BMI cutoffs for obesity in multi-ethnic populations by use of data on type 2 diabetes prevalence or a surrogate marker, small sample sizes, and self-reported disease status, including relatively few minority ethnic groups. Because type 2 diabetes can be delayed or prevented through dietary change, physical activity, and the early use of antihyperglycaemic therapy, it is important to establish BMI cutoffs for obesity in relation to the risk of type 2 diabetes among adults from minority ethnic populations in England that equate to those developed in White populations.

**Added value of this study**

In a comprehensive analysis, we define BMI cutoffs for obesity based on the risk of developing type 2 diabetes in minority ethnic adults equivalent to the BMI cutoff for obesity of 30·0 kg/m^2^ set for White populations. To our knowledge, this study is the first to provide BMI cutoffs for obesity for Arab populations and Black and south Asian ethnic subgroups. We also highlight the value of routine electronic health records and the use of large, linked datasets to provide precise ethnicity-specific BMI cutoffs for obesity. In our study, we included 1 472 819 people aged 18 years or older registered with a general practitioner practice in England at any point between 1990 and 2018 (1 333 816 were White, 75 956 were south Asian, 49 349 were Black, 10 934 were Chinese, and 2764 were Arab). For an equivalent age-adjusted and sex-adjusted incidence of type 2 diabetes at a BMI of 30·0 kg/m^2^ in White populations, we found lower BMI cutoffs for south Asian (23·9 kg/m^2^), Black (28·1 kg/m^2^), Chinese (26·9 kg/m^2^), and Arab (26·6 kg/m^2^) populations.

**Implications of all the available evidence**

By contrast to WHO expert consultation recommendations and NICE guidelines, our study shows that Black Caribbean, south Asian, Chinese, and Arab populations living in England had an equivalent risk of type 2 diabetes at substantially lower BMI values than the current BMI cutoffs for obesity. Our findings should guide revisions of current ethnicity-specific BMI cutoffs to trigger action to reduce the risk of developing type 2 diabetes and equalise opportunities for the increased prevention and early diagnosis of type 2 diabetes.

Several further attempts have been made to establish ethnicity-specific BMI cutoffs to identify obesity in relation to type 2 diabetes risk in multi-ethnic populations based in the UK and North America.^10^, ^11^, ^12^, ^13^ Such attempts had several limitations: the studies used prevalence data for type 2 diabetes^11^ or a surrogate marker,^12^ lacked precision because of small ethnic group sizes,^10^, ^11^, ^12^, ^13^ and did not examine particular minority ethnic groups.^10^, ^11^, ^12^, ^13^ To address these challenges, we used a large-scale, longitudinal database of linked primary and secondary care electronic health records from a representative sample of the population in England to identify BMI cutoffs for obesity based on the risk of developing type 2 diabetes among adults from Black, south Asian, Chinese, and Arab populations in England equivalent to the BMI obesity-related cutoff of 30·0 kg/m^2^ established in White populations.

## Methods

### Study design and participants

In this population-based cohort study, we used electronic health records across primary care (Clinical Practice Research Datalink [CPRD]) and hospital care (Hospital Episodes Statistics), with prospective recording and follow-up, which were linked by the CPRD and National Health Service (NHS) Digital by use of unique health-care identifiers. The CPRD is a real-world research service that collects anonymised patient data from a network of general practitioner practices (primary care clinics) across the UK. Nearly all (>99%) of the population in England is registered with a general practitioner practice. The CPRD is representative of the general population in England regarding sociodemographic characteristics and overall mortality.^14^

Eligible individuals were aged 18 years or older, without any past or current diagnosis of type 2 diabetes, and registered with a general practitioner practice in England at any point between Sept 1, 1990, and Dec 1, 2018, with at least 1 year of follow-up data. Individuals with a BMI of less than 15·0 kg/m^2^ and more than 50·0 kg/m^2^ or who were mixed race or part of an ethnic group other than White, Black, south Asian, Chinese, or Arab, had missing ethnicity data, or did not have a follow-up period of at least 1 year, were excluded.^15^ Ethical approval for the study was granted by the Independent Scientific Advisory Committee (19_035R) of the Medicines and Healthcare products Regulatory Agency in the UK in accordance with the Declaration of Helsinki. General practitioners do not need to seek individual patient consent when they share data with the CRPD.

### Procedures

We used CRPD data on age, sex, and self-reported smoking status at study entry. We used self-reported smoking status to classify individuals as either never smokers, ex-smokers, or current smokers. The 2015 English Index of Multiple Deprivation, based on practice location and divided according to quintiles, was used as the marker of socioeconomic position. Height (in cm) and weight (in kg) measurements are recorded in the CPRD whenever measured as part of routine primary care. In primary care, trained health-care staff typically measure height using a stadiometer and weight using class III weighing equipment. We used recorded BMI (kg/m^2^) attached to the BMI read code at study entry.

We assigned exposure as the earliest BMI recorded from the date of patients' registration at their current practice and the practices' up to standard date, or from the date at which the patient turned 18 years old. The up to standard date is the date at which the practice data is deemed to be of research quality. This date is derived using a CPRD algorithm that is primarily based on practice mortality recording and gaps in the data. To minimise reverse causality, we excluded the 12-month period following the date of the earliest BMI record from the risk period. If diagnoses of type 2 diabetes were recorded at the same time or soon after the BMI record, they could have influenced the BMI measurement because individuals with a new diagnosis of type 2 diabetes might adopt weight-control behaviours.

Patients with type 2 diabetes were identified by use of a CALIBER phenotyping algorithm.^16^, ^17^ This algorithm uses a combination of a general practitioner diagnosis of type 2 diabetes and hospital admissions data for patients who had a relevant diagnosis of type 2 diabetes (International Classification of Diseases, tenth revision). In the appendix (pp 2–17) are the code lists and a flowchart representation of diabetes phenotypes (appendix p 18). We only considered type 2 diabetes diagnoses after the earliest BMI record.

Self-reported ethnicity, identified by use of read codes recorded in the CPRD on ethnic group, country of origin, and language spoken, were collapsed into the 18 categories of the 2011 census of England and Wales and then into five categories (ie, White, Black, south Asian, Chinese, and Arab). We used self-reported ethnicity recorded in the CPRD where available and supplemented these data with self-reported ethnicity recorded in Hospital Episodes Statistics when required.^18^

### Statistical analysis

To identify BMI cutoffs for non-White minority ethnic populations that are risk-equivalent to a BMI of 30·0 kg/m^2^ for White populations, age-adjusted and sex-adjusted negative binomial regression models, with fractional polynomials for BMI, were fitted with incident type 2 diabetes and ethnicity data. To account for similarity of outcome within practices, statistical inference from the negative binomial models was based on cluster-robust SEs. We used a negative binomial model instead of a Poisson model to account for overdispersion.

We calculated the predicted age-adjusted and sex-adjusted incidence of type 2 diabetes among White individuals with a BMI of 30·0 kg/m^2^ (the established WHO-recommended and NICE-recommended cutoff used to define obesity). Back calculations were done to obtain ethnicity-specific BMI cutoffs (appendix p 19). Using the same model, we calculated the BMI value for the predicted incidence of type 2 diabetes among individuals from Black, south Asian, Chinese, and Arab ethnic populations. We repeated analyses for Black ethnic subgroups (ie, Black Africans, Black Caribbean, Black British, and other Black people) and south Asian ethnic subgroups (ie, Indian, Pakistani, Bangladeshi, Nepali, Sri Lankan, and Tamil). Given that WHO's classification of obesity according to BMI is age-independent and sex-independent, we only adjusted for age and sex in the main analyses. We repeated the analyses with adjustments for smoking status and socioeconomic position (as measured by the Index of Multiple Deprivation). Using the same methods as described, we also identified BMI cutoffs for Black, south Asian, Chinese, and Arab ethnic populations that are risk-equivalent to a BMI of 25·0 kg/m^2^ for White populations (the established WHO-recommended and NICE-recommended cutoff used to define overweight).

95% CIs around predicted BMI cutoffs for obesity were estimated by use of an approach similar to the fiducial approach.^19^ This method involved identifying a corresponding upper and lower CI around the BMI cutoff on the basis of the respective upper and lower CIs for type 2 diabetes incidence.

Considering that routinely recorded data in primary care electronic health records are likely to be missing in a manner that is not random, multiple imputation of missing data was considered inappropriate because the assumption of missing at random was unlikely to be met. Therefore, a complete case analysis approach was used.^20^ All analyses were done by use of Stata, version 16.0.

### Role of the funding source

The funder of the study had no role in study design, data collection, data analysis, data interpretation, or writing of the report.

## Results

**Figure 1:**
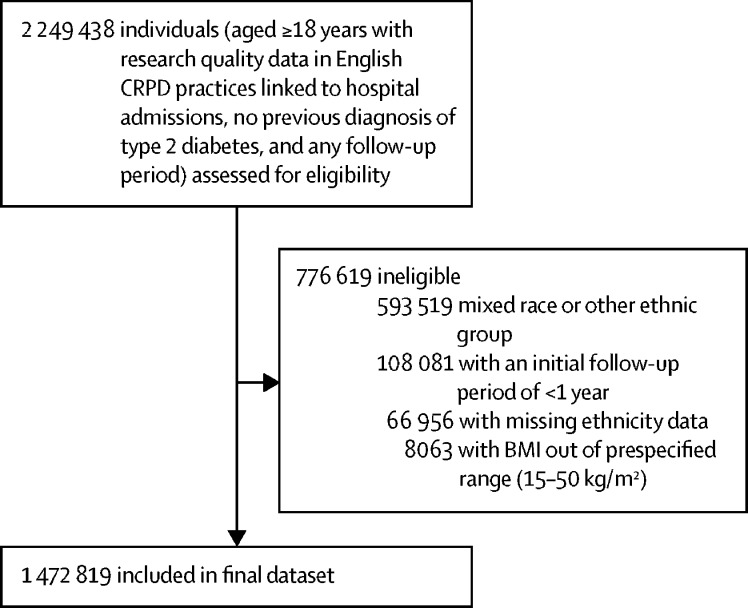
Study profile CRPD=Clinical Practice Research Datalink.

From a total of 2 249 438 individuals aged 18 years or older with no previous diagnosis of diabetes and with any follow-up, recruited between Sept 1, 1990, and Dec 1, 2018, 1 472 819 were included in the study (figure 1). 1 333 816 (90·6%) participants were White, 75 956 (5·2%) were south Asian, 49 349 (3·4%) were Black, 10 934 (0·7%) were Chinese, and 2764 (0·2%) were Arab (table; appendix p 20). More women than men were included in the study, and, at baseline, the mean BMI was lowest among the Chinese group than among the other ethnic groups (table).

**Table:** Baseline characteristics of the study population by ethnicity

		**Total (n=1 472 819)**	**White (n=1 333 816)**	**South Asian (n=75 956)**	**Black (n=49 349)**	**Chinese (n=10 934)**	**Arab (n=2764)**
Age, years		44·9 (17·9)	45·7 (18·2)	36·6 (13·3)	37·8 (12·9)	36·7 (13·2)	36·8 (12·6)
Sex							
	Female	846 355 (57·5%)	770 203 (57·7%)	39 387 (51·9%)	28 688 (58·1%)	6769 (61·9%)	1308 (47·3%)
	Male	626 464 (42·5%)	563 613 (42·3%)	36 569 (48·1%)	20 661 (41·9%)	4165 (38·1%)	1456 (52·7%)
Smoking status							
	Never smoker	754 436 (51·2%)	648 609 (48·6%)	58 415 (76·9%)	37 317 (75·6%)	8262 (75·6%)	1833 (66·3%)
	Ex-smoker	378 414 (25·7%)	365 456 (27·4%)	6560 (8·6%)	4965 (10·1%)	1120 (10·2%)	313 (11·3%)
	Current smoker	339 969 (23·1%)	319 751 (24·0%)	10 981 (14·5%)	7067 (14·3%)	1552 (14·2%)	618 (22·4%)
Index of Multiple Deprivation*							
	1	197 039 (13·4%)	184 600 (13·8%)	8240 (10·8%)	2385 (4·8%)	1598 (14·6%)	216 (7·8%)
	2	278 783 (18·9%)	259 891 (19·5%)	11 739 (15·5%)	4667 (9·5%)	2180 (19·9%)	306 (11·1%)
	3	297 211 (20·2%)	272 263 (20·4%)	15 700 (20·7%)	6787 (13·8%)	1882 (17·2%)	579 (20·9%)
	4	328 359 (22·3%)	290 449 (21·8%)	19 588 (25·8%)	15 077 (30·6%)	2417 (22·1%)	828 (30·0%)
	5	371 427 (25·2%)	326 613 (24·5%)	20 689 (27·2%)	20 433 (41·4%)	2857 (26·1%)	835 (30·2%)
BMI, kg/m^2^		26·1 (5·1)	26·2 (5·1)	25·1 (4·6)	27·1 (5·4)	22·6 (3·7)	26·5 (5·0)

Data are mean (SD) or n (%).

* The first quintile represents the least deprived and the fifth quintile represents the most deprived.

After a median follow-up of 6·5 years (IQR 3·2–11·2), 97 823 (6·6%) of the 1 472 819 individuals in our study were diagnosed with type 2 diabetes. Of the 97 823 diagnosed participants, 89 287 (91·3%) were White, 5632 (5·8%) were south Asian, 2444 (2·5%) were Black, 317 (0·3%) were Chinese, and 143 (0·1%) were Arab. The median age at diagnosis of type 2 diabetes was 67 years (IQR 57–76) in White individuals, 55 years (45–65) in south Asian individuals, 54 years (47–65) in Black individuals, 60 years (52–68) in Chinese individuals, and 56 years (47–64) in Arab individuals.

**Figure 2:**
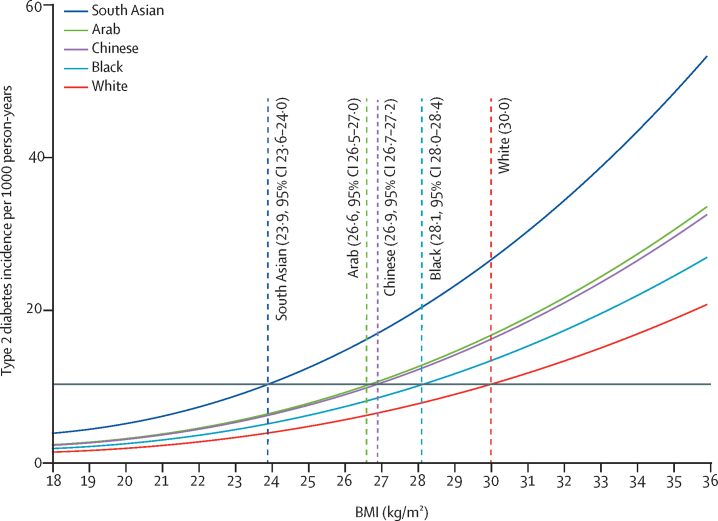
Age-adjusted and sex-adjusted BMI cutoffs in minority ethnic populations in England equivalent to a BMI cutoff of 30·0 kg/m^2^ in White populations in relation to type 2 diabetes incidence The incidence of type 2 diabetes for a BMI of 30·0 kg/m^2^ in the White population can be read off the graph at the intersection of the grey horizontal line and the fitted line for the White population.

**Figure 3:**
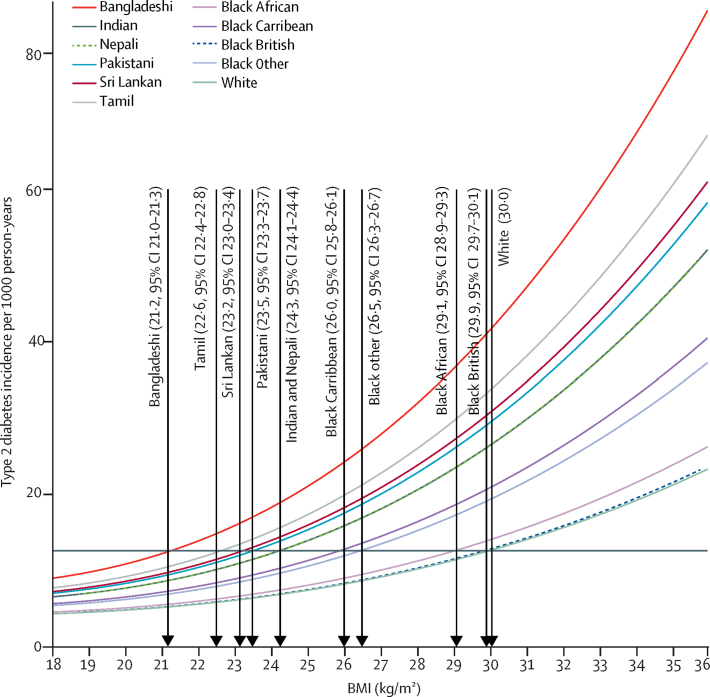
Age-adjusted and sex-adjusted BMI cutoffs in minority ethnic subgroups in England equivalent to a BMI cutoff of 30·0 kg/m^2^ in White populations in relation to type 2 diabetes incidence The incidence of type 2 diabetes for a BMI of 30·0 kg/m^2^ in the White population can be read off the graph at the intersection of the grey horizontal line and the fitted line for the White population.

For the equivalent age-adjusted and sex-adjusted incidence of type 2 diabetes at a BMI of 30·0 kg/m^2^ in White populations, the BMI cutoffs were 23·9 kg/m^2^ (95% CI 23·6–24·0) in south Asian populations, 28·1 kg/m^2^ (28·0–28·4) in Black populations, 26·9 kg/m^2^ (26·7–27·2) in Chinese populations, and 26·6 kg/m^2^ (26·5–27·0) in Arab populations (figure 2). Further adjustment for self-reported smoking status and socioeconomic position did not substantially change the estimated ethnicity-specific BMI cutoffs for obesity (appendix p 21). The BMI cutoffs for Black ethnic subgroups and south Asian ethnic subgroups equivalent to a BMI of 30·0 kg/m^2^ for White populations, related to the age-adjusted and sex-adjusted incidence of type 2 diabetes, can be found in figure 3.

**Figure 4:**
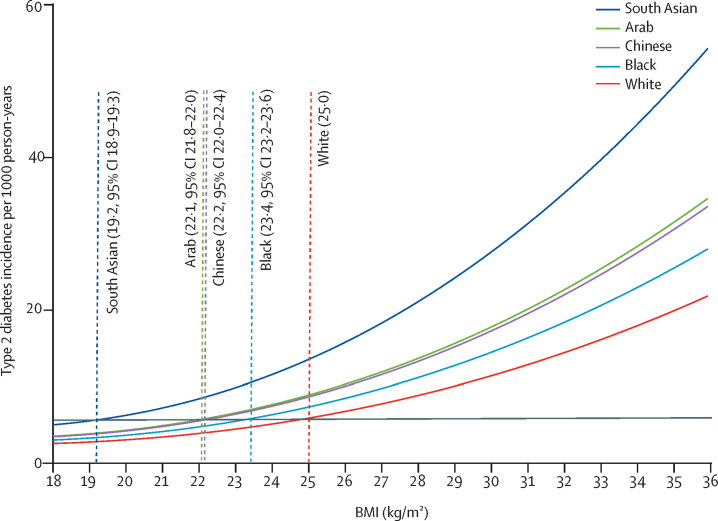
Age-adjusted and sex-adjusted BMI cutoffs in minority ethnic populations in England equivalent to a BMI cutoff of 25·0 kg/m^2^ in White populations in relation to type 2 diabetes incidence The incidence of type 2 diabetes for a BMI of 25·0 kg/m^2^ in the White population can be read off the graph at the intersection of the grey horizontal line and the fitted line for the White population.

For the equivalent age-adjusted and sex-adjusted incidence of type 2 diabetes at a BMI of 25·0 kg/m^2^ in White populations, the BMI cutoffs were 19·2 kg/m^2^ (95% CI 18·9–19·3) in south Asian populations, 23·4 kg/m^2^ (23·2–23·6) in Black populations, 22·2 kg/m^2^ (22·0–22·4) in Chinese populations, and 22·1 kg/m^2^ (21·8–22·0) in Arab populations (figure 4).

## Discussion

Using electronic health records from approximately 1·5 million individuals, of whom 97 823 were diagnosed with type 2 diabetes during a median follow-up of 6·5 years, we provide new BMI thresholds for obesity to trigger action to reduce the risk of developing type 2 diabetes in Black, south Asian, Chinese, and Arab populations living in England. Our data address the ongoing debate around the interpretation of recommended BMI cutoffs for identifying obesity in minority ethnic populations. For an equivalent age-adjusted and sex-adjusted incidence of type 2 diabetes at a BMI of 30·0 kg/m^2^ in White populations, we found lower BMI cutoffs for south Asian (23·9 kg/m^2^), Black (28·1 kg/m^2^), Chinese (26·9 kg/m^2^), and Arab (26·6 kg/m^2^) populations.

Obesity, defined as a BMI of 30·0 kg/m^2^ or more, is a widely used measure and an important risk factor for the development of type 2 diabetes.^21^ However, the appropriateness of this BMI cutoff in non-White minority ethnic populations is contentious, and remains a subject of debate because of important limitations in the evidence base.^3^, ^4^, ^22^, ^23^ Previously reported studies that attempted to identify BMI cutoffs for obesity in multi-ethnic populations relied on prevalence data,^12^ in which BMI and type 2 diabetes status were ascertained at the same timepoint, lacked precision because of small sample sizes,^10^, ^12^, ^13^ used surrogate markers^12^ and self-reported data,^10^, ^11^ and included relatively few ethnic groups.^13^

WHO and NICE both recommend a BMI cutoff of 27·5 kg/m^2^ to define obesity in south Asian and Chinese populations to trigger lifestyle interventions.^3^, ^4^ NICE also suggest that this lower BMI threshold should be used to trigger action to prevent type 2 diabetes among Black populations.^24^ Our study clearly showed that, compared with the risk of developing type 2 diabetes at a BMI of 30·0 kg/m^2^ in White populations, the equivalent risk among south Asian individuals occurred at a BMI of 23·9 kg/m^2^, a cutoff much lower than the recommended ethnicity-specific cutoff of 27·5 kg/m^2^. Our findings are consistent with previous studies in suggesting that the cutoffs currently recommended by WHO and NICE should be reduced when applied to non-White populations.^10^, ^11^, ^12^, ^13^ For example, in the SABRE (Southall and Brent Revisited) cohort study^13^ of Europeans (n=1356), south Asians (n=842), and African-Caribbeans (n=335) in north and west London, UK, aged 40–69 years at baseline (recruited between 1988 and 1991) and followed up for a median of 19 years, age-adjusted and sex-adjusted BMI cutoffs for obesity were 25·2 kg/m^2^ for south Asians and 27·2 kg/m^2^ for African-Caribbeans. We found that the incidences of type 2 diabetes among south Asian subpopulations (ie, Indian, Pakistani, Bangladeshi, Nepali, Sri Lankan, and Tamil) were equivalent to that in the White population at consistently much lower values of BMI. However, when examining Black ethnic subgroups (ie, Black Africans, Black Caribbean, Black British, and other Black people), we found that the incidences of type 2 diabetes were equivalent to that in the White population at lower BMI values only for Black Caribbean individuals and Black people of other ethnic origins. We also found that BMI cutoffs for overweight based on the risk of type 2 diabetes were lower for south Asian, Black, Chinese, and Arab populations than for White populations (25·0 kg/m^2^), suggesting that the recommended BMI cutoff for overweight to trigger action to reduce the risk of type 2 diabetes should also be lowered in these groups. Whether lower BMI cutoffs in non-White populations are due to differences in body composition, biochemical characteristics, lifestyle factors (eg, physical activity or diet), the genetic architecture of type 2 diabetes, or lifestyle–gene interactions remains unclear.^25^, ^26^, ^27^ Future studies that examine the relative contributions of these mechanisms to the development of type 2 diabetes might help to explain our study findings.

A limitation of our study is that, even though individuals registered in the CPRD are representative of the general adult population in the UK,^14^ individuals with a recorded BMI measurement might not necessarily be representative of the general UK population. BMI data, if not recorded as part of registration with a general practitioner, tends to be recorded opportunistically (ie, when the patient is using health-care services for other reasons or when a BMI measurement is of direct clinical importance). We reduced this possibility by only using the first BMI value recorded from the registration date (these values would have probably been recorded for administrative and not health reasons). Our findings of lower BMI cutoffs for obesity in minority ethnic populations compared with White populations living in England apply only to the risk of developing type 2 diabetes, and might not apply to other endpoints, such as cardiovascular disease or all-cause mortality. Type 2 diabetes was chosen as the outcome of interest because it is the most specific obesity-related complication and a chronic, progressive disease with considerable health and socioeconomic costs.^21^ BMI is a simple, inexpensive surrogate measure of body fat used in primary and secondary care, and is the subject of national and international guidelines on assessing adiposity.^3^, ^4^, ^28^ Unlike BMI, body composition measures (eg, waist to hip ratio and total body fat) are not routinely measured in primary or secondary care, but might help to explain differences in the risk of type 2 diabetes between populations. In primary care, height and weight measurements are recorded as part of routine care by trained staff using medical grade anthropometric equipment. However, we were unable to verify whether all the individuals were measured in a similar and uniform manner across the practices over the 28 years between 1990 and 2018. The generational status of minority ethnic populations in electronic health records in primary and secondary care is not recorded. Therefore, we cannot be certain of the extent to which our findings are applicable to future generations or to minority ethnic populations living in other countries or their country of origin.

A major strength of our study is the large sample size drawn from English primary care electronic health records, with linkage to secondary care records. This large sample size enabled us to reliably estimate BMI cutoffs for overweight and obesity for the four main minority ethnic populations currently living in England. The inclusion of the Arab population and the sufficiently granular categorisation of Black and south Asian individuals into ethnic subgroups is instructive, permitting clinicians to manage health-care needs related to these ethnic groups at much lower BMI values. Ascertainment of type 2 diabetes diagnoses from electronic health records was by a validated algorithm designed to minimise miscoding and misclassification of diabetes type, thereby reducing the likelihood that individuals with type 1 diabetes or other forms of diabetes were included in our study population.^17^ Restriction of the study sample to individuals with at least 12 months of continuous registration before their initial diagnosis of type 2 diabetes ensured that diagnoses were truly incident. By restricting our analyses to individuals of White, Black, south Asian, Chinese, and Arab populations, we were able to make clinically relevant comparisons between well defined populations with distinct biological, sociocultural, and demographic characteristics. This approach facilitated the meaningful characterisation of ethnicity. Linkage to area-level deprivation data additionally enabled us to separate the influences of ethnicity and socioeconomic deprivation, factors that frequently conflate. By adjusting for the clustering of individuals within general practices with robust SEs, we attempted to account for the influence of practice-level factors on BMI cutoffs. To optimise the coverage of ethnicity, we used an ontological approach to incorporate codes referring to country of origin and language spoken, in addition to codes for ethnic group, all of which convey some information about ethnicity.^29^

Improving and optimising access to weight management services and therapies for individuals belonging to minority ethnic populations would facilitate the population-based management of obesity. However, access to weight management services in England is largely determined by BMI cutoffs, which, as our study shows, are inadequate for establishing the risk of type 2 diabetes among non-White minority ethnic populations. The choice of a BMI cutoff for obesity related to type 2 diabetes risk has a profound effect on patient care, including the referral of patients to weight management services, opportunistic and proactive screening for type 2 diabetes within the population, and raising awareness more generally. Currently, WHO-recommended and NICE-recommended BMI cutoffs for obesity do not provide an adequate basis for taking action. NICE's recommendation for preventing type 2 diabetes among Asian (south Asian and Chinese) populations uses the lower BMI threshold of 27·5 kg/m^2^ to indicate the risk for type 2 diabetes equivalent to a BMI of 30·0 kg/m^2^ in White populations. NICE have also indicated that this lower BMI threshold of 27·5 kg/m^2^ for Asian populations should be used to trigger action to prevent type 2 diabetes among Black populations. Conversely, our data reveal various BMI cutoffs to identify obesity based on type 2 diabetes risk among Black ethnic subgroups. There are well established and effective lifestyle strategies for the prevention, and to delay the onset, of type 2 diabetes for those at risk.^5^ Furthermore, optimised glycaemic control facilitates the prevention and reduced progression of the longer-term complications of type 2 diabetes.^6^, ^7^, ^8^, ^9^ However, without effective prevention and early diagnosis of type 2 diabetes, these advantages cannot be fully realised. The risk of type 2 diabetes for several minority ethnic groups is under-recognised due to the existing BMI cutoff criteria recommended by WHO and NICE. This under-recognition could hinder opportunities for the increased prevention and early diagnosis of type 2 diabetes for patients in these minority ethnic groups. It is therefore crucial to revise the classification of obesity among minority ethnic populations using BMI cutoffs that more appropriately apply to them.

To conclude, we provide compelling data to support a complete revision of the BMI cutoffs currently used to trigger action to prevent type 2 diabetes in England. Such a revision should ensure the provision of appropriate clinical surveillance for patients in minority ethnic populations, commensurate with their greater risk of type 2 diabetes, that would help to prevent the future onset, and therefore facilitate early and effective treatment, of type 2 diabetes. Further research is required to examine whether the same ethnicity-specific BMI cutoffs for overweight and obesity can be applied to trigger action to prevent type 2 diabetes in other countries.

**This online publication has been corrected. The corrected version first appeared at thelancet.com/diabetes-endocrinology on June 16, 2021**

## Data sharing

Data from this study will not be made available because accessing patient-level data from the CPRD requires an application and permissions.

## Declaration of interests

PG is a trustee of the South Asian Health Foundation, is part funded by the National Institute for Health Research (NIHR) Applied Research Collaboration West Midlands, and is a NIHR senior investigator. All other authors declare no competing interests.
