# Correction to *Lancet Diabetes Endocrinol* 2021; 9: 419–26

**DOI:** 10.1016/S2213-8587(21)00151-0

**Published:** 2021-07

*Caleyachetty R, Barber TM, Mohammed NI, et al. Ethnicity-specific BMI cutoffs for obesity based on type 2 diabetes risk in England: a population-based cohort study.* Lancet Diabetes Endocrinol *2021; **9:** 419–26*—In figure 4 of this Article, the horizontal line representing type 2 diabetes incidence at a BMI of 25·0 kg/m^2^ in White populations was incorrectly placed. This correction has been made to the online version as of June 16, 2021, and printed version is correct.

