# Supplementary material for: Ethnicity-specific BMI cutoffs for obesity based on type 2 diabetes risk in England: a population-based cohort study
Source: Lancet Diabetes Endocrinol. 2021 Jul;9(7):419–26. doi: 10.1016/S2213-8587(21)00088-7 (PMC8208895; doi:10.1016/S2213-8587(21)00088-7)

THE LANCET
Diabetes & Endocrinology

Supplementary appendix

This appendix formed part of the original submission and has been peer reviewed. We post it as supplied by the authors.

Supplement to: Caleyachetty R, Barber T M, Mohammed N I, et al. Ethnicity-specific BMI cutoffs for obesity based on type 2 diabetes risk in England: a population-based cohort study. *Lancet Diabetes Endocrinol* 2021; published online May 11. https://doi.org/10.1016/S2213-8587(21)00088-7.

Supplementary Appendix

Table S1. Clinical code list from primary care EHR data source..2

Table S2. ICD-10 code list from secondary care EHR data source..17

Figure S1. Diabetes flowchart representation 18

Calculating ethnic-specific BMI cut-off points………………………………………………………………..19

Table S3. Ethnic breakdown of study population...20

Figure S2. Type 2 diabetes mellitus incidence rates during median 6.5 years follow-up by baseline body mass index among main ethnic groups in England. Negative binomial regression model adjusted for age, sex, smoking status and social deprivation…………………………………………………………………………21

Table S1. Clinical code list from primary care EHR data source

Category (code)	Clinical code	Clinical term
Type I diabetes mellitus (3)	C108.12	Type 1 diabetes mellitus
Type I diabetes mellitus (3)	C108.13	Type I diabetes mellitus
Type I diabetes mellitus (3)	C108011	Type I diabetes mellitus with renal complications
Type I diabetes mellitus (3)	C108012	Type 1 diabetes mellitus with renal complications
Type I diabetes mellitus (3)	C108211	Type I diabetes mellitus with neurological complications
Type I diabetes mellitus (3)	C108212	Type 1 diabetes mellitus with neurological complications
Type I diabetes mellitus (3)	C108411	Unstable type I diabetes mellitus
Type I diabetes mellitus (3)	C108412	Unstable type 1 diabetes mellitus
Type I diabetes mellitus (3)	C108511	Type I diabetes mellitus with ulcer
Type I diabetes mellitus (3)	C108512	Type 1 diabetes mellitus with ulcer
Type I diabetes mellitus (3)	C108711	Type I diabetes mellitus with retinopathy
Type I diabetes mellitus (3)	C108712	Type 1 diabetes mellitus with retinopathy
Type I diabetes mellitus (3)	C108811	Type I diabetes mellitus - poor control
Type I diabetes mellitus (3)	C108812	Type 1 diabetes mellitus - poor control
Type I diabetes mellitus (3)	C108911	Type I diabetes mellitus maturity onset
Type I diabetes mellitus (3)	C108912	Type 1 diabetes mellitus maturity onset
Type I diabetes mellitus (3)	C108A11	Type I diabetes mellitus without complication
Type I diabetes mellitus (3)	C108D11	Type I diabetes mellitus with nephropathy
Type I diabetes mellitus (3)	C108E11	Type I diabetes mellitus with hypoglycaemic coma
Type I diabetes mellitus (3)	C108E12	Type 1 diabetes mellitus with hypoglycaemic coma
Type I diabetes mellitus (3)	C108F11	Type I diabetes mellitus with diabetic cataract
Type I diabetes mellitus (3)	C108H11	Type I diabetes mellitus with arthropathy
Type I diabetes mellitus (3)	C108J11	Type I diabetes mellitus with neuropathic arthropathy
Type I diabetes mellitus (3)	C108J12	Type 1 diabetes mellitus with neuropathic arthropathy
Type I diabetes mellitus (3)	C10E.00	Type 1 diabetes mellitus
Type I diabetes mellitus (3)	C10E.11	Type I diabetes mellitus
Type I diabetes mellitus (3)	C10E000	Type 1 diabetes mellitus with renal complications
Type I diabetes mellitus (3)	C10E100	Type 1 diabetes mellitus with ophthalmic complications
Type I diabetes mellitus (3)	C10E200	Type 1 diabetes mellitus with neurological complications
Type I diabetes mellitus (3)	C10E300	Type 1 diabetes mellitus with multiple complications
Type I diabetes mellitus (3)	C10E311	Type I diabetes mellitus with multiple complications
Type I diabetes mellitus (3)	C10E400	Unstable type 1 diabetes mellitus
Type I diabetes mellitus (3)	C10E411	Unstable type I diabetes mellitus
Type I diabetes mellitus (3)	C10E500	Type 1 diabetes mellitus with ulcer
Type I diabetes mellitus (3)	C10E511	Type I diabetes mellitus with ulcer
Type I diabetes mellitus (3)	C10E600	Type 1 diabetes mellitus with gangrene
Type I diabetes mellitus (3)	C10E700	Type 1 diabetes mellitus with retinopathy
Type I diabetes mellitus (3)	C10E711	Type I diabetes mellitus with retinopathy
Type I diabetes mellitus (3)	C10E800	Type 1 diabetes mellitus - poor control
Type I diabetes mellitus (3)	C10E900	Type 1 diabetes mellitus maturity onset
Type I diabetes mellitus (3)	C10E911	Type I diabetes mellitus maturity onset
Type I diabetes mellitus (3)	C10EA00	Type 1 diabetes mellitus without complication
Type I diabetes mellitus (3)	C10EA11	Type I diabetes mellitus without complication
Type I diabetes mellitus (3)	C10EB00	Type 1 diabetes mellitus with mononeuropathy
Type I diabetes mellitus (3)	C10EC00	Type 1 diabetes mellitus with polyneuropathy

Type I diabetes mellitus (3)	C10EC11	Type I diabetes mellitus with polyneuropathy
Type I diabetes mellitus (3)	C10ED00	Type 1 diabetes mellitus with nephropathy
Type I diabetes mellitus (3)	C10EE00	Type 1 diabetes mellitus with hypoglycaemic coma
Type I diabetes mellitus (3)	C10EF00	Type 1 diabetes mellitus with diabetic cataract
Type I diabetes mellitus (3)	C10EG00	Type 1 diabetes mellitus with peripheral angiopathy
Type I diabetes mellitus (3)	C10EH00	Type 1 diabetes mellitus with arthropathy
Type I diabetes mellitus (3)	C10EJ00	Type 1 diabetes mellitus with neuropathic arthropathy
Type I diabetes mellitus (3)	C10EK00	Type 1 diabetes mellitus with persistent proteinuria
Type I diabetes mellitus (3)	C10EL00	Type 1 diabetes mellitus with persistent microalbuminuria
Type I diabetes mellitus (3)	C10EM00	Type 1 diabetes mellitus with ketoacidosis
Type I diabetes mellitus (3)	C10EM11	Type I diabetes mellitus with ketoacidosis
Type I diabetes mellitus (3)	C10EN00	Type 1 diabetes mellitus with ketoacidotic coma
Type I diabetes mellitus (3)	C10EN11	Type I diabetes mellitus with ketoacidotic coma
Type I diabetes mellitus (3)	C10EP00	Type 1 diabetes mellitus with exudative maculopathy
Type I diabetes mellitus (3)	C10EP11	Type I diabetes mellitus with exudative maculopathy
Type I diabetes mellitus (3)	C10EQ00	Type 1 diabetes mellitus with gastroparesis
Type II diabetes mellitus (4)	C109.12	Type 2 diabetes mellitus
Type II diabetes mellitus (4)	C109.13	Type II diabetes mellitus
Type II diabetes mellitus (4)	C109011	Type II diabetes mellitus with renal complications
Type II diabetes mellitus (4)	C109012	Type 2 diabetes mellitus with renal complications
Type II diabetes mellitus (4)	C109111	Type II diabetes mellitus with ophthalmic complications
Type II diabetes mellitus (4)	C109112	Type 2 diabetes mellitus with ophthalmic complications
Type II diabetes mellitus (4)	C109211	Type II diabetes mellitus with neurological complications
Type II diabetes mellitus (4)	C109212	Type 2 diabetes mellitus with neurological complications
Type II diabetes mellitus (4)	C109411	Type II diabetes mellitus with ulcer
Type II diabetes mellitus (4)	C109412	Type 2 diabetes mellitus with ulcer
Type II diabetes mellitus (4)	C109511	Type II diabetes mellitus with gangrene
Type II diabetes mellitus (4)	C109512	Type 2 diabetes mellitus with gangrene
Type II diabetes mellitus (4)	C109611	Type II diabetes mellitus with retinopathy
Type II diabetes mellitus (4)	C109612	Type 2 diabetes mellitus with retinopathy
Type II diabetes mellitus (4)	C109711	Type II diabetes mellitus - poor control
Type II diabetes mellitus (4)	C109712	Type 2 diabetes mellitus - poor control
Type II diabetes mellitus (4)	C109A11	Type II diabetes mellitus with mononeuropathy
Type II diabetes mellitus (4)	C109B11	Type II diabetes mellitus with polyneuropathy
Type II diabetes mellitus (4)	C109C11	Type II diabetes mellitus with nephropathy
Type II diabetes mellitus (4)	C109C12	Type 2 diabetes mellitus with nephropathy
Type II diabetes mellitus (4)	C109D11	Type II diabetes mellitus with hypoglycaemic coma
Type II diabetes mellitus (4)	C109D12	Type 2 diabetes mellitus with hypoglycaemic coma
Type II diabetes mellitus (4)	C109E11	Type II diabetes mellitus with diabetic cataract
Type II diabetes mellitus (4)	C109E12	Type 2 diabetes mellitus with diabetic cataract
Type II diabetes mellitus (4)	C109F11	Type II diabetes mellitus with peripheral angiopathy
Type II diabetes mellitus (4)	C109F12	Type 2 diabetes mellitus with peripheral angiopathy
Type II diabetes mellitus (4)	C109G11	Type II diabetes mellitus with arthropathy
Type II diabetes mellitus (4)	C109G12	Type 2 diabetes mellitus with arthropathy
Type II diabetes mellitus (4)	C109H11	Type II diabetes mellitus with neuropathic arthropathy
Type II diabetes mellitus (4)	C109H12	Type 2 diabetes mellitus with neuropathic arthropathy

Type II diabetes mellitus (4)	C109J00	Insulin treated Type 2 diabetes mellitus
Type II diabetes mellitus (4)	C109J12	Insulin treated Type II diabetes mellitus
Type II diabetes mellitus (4)	C109K00	Hyperosmolar non-ketotic state in type 2 diabetes mellitus
Type II diabetes mellitus (4)	C10F.00	Type 2 diabetes mellitus
Type II diabetes mellitus (4)	C10F.11	Type II diabetes mellitus
Type II diabetes mellitus (4)	C10F000	Type 2 diabetes mellitus with renal complications
Type II diabetes mellitus (4)	C10F011	Type II diabetes mellitus with renal complications
Type II diabetes mellitus (4)	C10F100	Type 2 diabetes mellitus with ophthalmic complications
Type II diabetes mellitus (4)	C10F200	Type 2 diabetes mellitus with neurological complications
Type II diabetes mellitus (4)	C10F211	Type II diabetes mellitus with neurological complications
Type II diabetes mellitus (4)	C10F300	Type 2 diabetes mellitus with multiple complications
Type II diabetes mellitus (4)	C10F311	Type II diabetes mellitus with multiple complications
Type II diabetes mellitus (4)	C10F400	Type 2 diabetes mellitus with ulcer
Type II diabetes mellitus (4)	C10F411	Type II diabetes mellitus with ulcer
Type II diabetes mellitus (4)	C10F500	Type 2 diabetes mellitus with gangrene
Type II diabetes mellitus (4)	C10F600	Type 2 diabetes mellitus with retinopathy
Type II diabetes mellitus (4)	C10F611	Type II diabetes mellitus with retinopathy
Type II diabetes mellitus (4)	C10F700	Type 2 diabetes mellitus - poor control
Type II diabetes mellitus (4)	C10F711	Type II diabetes mellitus - poor control
Type II diabetes mellitus (4)	C10F900	Type 2 diabetes mellitus without complication
Type II diabetes mellitus (4)	C10F911	Type II diabetes mellitus without complication
Type II diabetes mellitus (4)	C10FA00	Type 2 diabetes mellitus with mononeuropathy
Type II diabetes mellitus (4)	C10FA11	Type II diabetes mellitus with mononeuropathy
Type II diabetes mellitus (4)	C10FB00	Type 2 diabetes mellitus with polyneuropathy
Type II diabetes mellitus (4)	C10FB11	Type II diabetes mellitus with polyneuropathy
Type II diabetes mellitus (4)	C10FC00	Type 2 diabetes mellitus with nephropathy
Type II diabetes mellitus (4)	C10FD00	Type 2 diabetes mellitus with hypoglycaemic coma
Type II diabetes mellitus (4)	C10FD11	Type II diabetes mellitus with hypoglycaemic coma
Type II diabetes mellitus (4)	C10FE00	Type 2 diabetes mellitus with diabetic cataract
Type II diabetes mellitus (4)	C10FE11	Type II diabetes mellitus with diabetic cataract
Type II diabetes mellitus (4)	C10FF00	Type 2 diabetes mellitus with peripheral angiopathy
Type II diabetes mellitus (4)	C10FG00	Type 2 diabetes mellitus with arthropathy
Type II diabetes mellitus (4)	C10FH00	Type 2 diabetes mellitus with neuropathic arthropathy
Type II diabetes mellitus (4)	C10FJ00	Insulin treated Type 2 diabetes mellitus
Type II diabetes mellitus (4)	C10FJ11	Insulin treated Type II diabetes mellitus
Type II diabetes mellitus (4)	C10FK00	Hyperosmolar non-ketotic state in type 2 diabetes mellitus
Type II diabetes mellitus (4)	C10FL00	Type 2 diabetes mellitus with persistent proteinuria
Type II diabetes mellitus (4)	C10FL11	Type II diabetes mellitus with persistent proteinuria
Type II diabetes mellitus (4)	C10FM00	Type 2 diabetes mellitus with persistent microalbuminuria
Type II diabetes mellitus (4)	C10FM11	Type II diabetes mellitus with persistent microalbuminuria
Type II diabetes mellitus (4)	C10FN00	Type 2 diabetes mellitus with ketoacidosis
Type II diabetes mellitus (4)	C10FP00	Type 2 diabetes mellitus with ketoacidotic coma
Type II diabetes mellitus (4)	C10FQ00	Type 2 diabetes mellitus with exudative maculopathy
Type II diabetes mellitus (4)	C10FR00	Type 2 diabetes mellitus with gastroparesis
H/O diabetes (1)	1434	H/O: diabetes mellitus

H/O diabetes (1)	14F4.00	H/O: Admission in last year for diabetes foot problem
H/O diabetes (1)	14P3.00	H/O: insulin therapy
H/O diabetes (1)	2126300	Diabetes resolved
H/O diabetes (1)	212H.00	Diabetes resolved
H/O diabetes (1)	9OL9.00	Diabetes monitoring deleted
Possible diabetes (2)	13Y1.00	Diabetic association member
Possible diabetes (2)	3881	Education score - diabetes
Possible diabetes (2)	3882	Diabetes well being questionnaire
Possible diabetes (2)	66A..00	Diabetic monitoring
Possible diabetes (2)	66A1.00	Initial diabetic assessment
Possible diabetes (2)	66A2.00	Follow-up diabetic assessment
Possible diabetes (2)	66AM.00	Diabetic - follow-up default
Possible diabetes (2)	66AZ.00	Diabetic monitoring NOS
Possible diabetes (2)	66Af.00	Patient diabetes education review
Possible diabetes (2)	66Ak.00	Diabetic monitoring - lower risk albumin excretion
Possible diabetes (2)	66Al.00	Diabetic monitoring - higher risk albumin excretion
Possible diabetes (2)	679L.00	Health education - diabetes
Possible diabetes (2)	679R.00	Patient offered diabetes structured education programme
Possible diabetes (2)	8A12.00	Diabetic crisis monitoring
Possible diabetes (2)	8A17.00	Self monitoring of blood glucose
Possible diabetes (2)	8A18.00	Self monitoring of urine glucose
Possible diabetes (2)	8A19.00	Self monitoring of blood and urine glucose
Possible diabetes (2)	8A1A.00	Self monitoring urine ketones
Possible diabetes (2)	8CR2.00	Diabetes clinical management plan
Possible diabetes (2)	8CS0.00	Diabetes care plan agreed
Possible diabetes (2)	8HHy.00	Referral to diabetic register
Possible diabetes (2)	8HTe.00	Referral to diabetes preconception counselling clinic
Possible diabetes (2)	8HTk.00	Referral to diabetic eye clinic
Possible diabetes (2)	8Hg4.00	Discharged from care of diabetes specialist nurse
Possible diabetes (2)	8Hj0.00	Referral to diabetes structured education programme
Possible diabetes (2)	8Hj3.00	Referral to DAFNE diabetes structured education programme
Possible diabetes (2)	8Hj4.00	Referral to DESMOND diabetes structured education programme
Possible diabetes (2)	8Hj5.00	Referral to XPERT diabetes structured education programme
Possible diabetes (2)	8I6F.00	Diabetic retinopathy screening not indicated
Possible diabetes (2)	8I6G.00	Diabetic foot examination not indicated
Possible diabetes (2)	8I81.00	Did not complete diabetes structured education programme
Possible diabetes (2)	8I82.00	Did not complete DAFNE diabetes structured education program
Possible diabetes (2)	8I83.00	Did not complete DESMOND diabetes structured educat program
Possible diabetes (2)	8I84.00	Did not complete XPERT diabetes structured education program
Possible diabetes (2)	93C4.00	Patient consent given for addition to diabetic register
Possible diabetes (2)	9N0m.00	Seen in diabetic nurse consultant clinic
Possible diabetes (2)	9N0n.00	Seen in community diabetes specialist clinic
Possible diabetes (2)	9N0o.00	Seen in community diabetic specialist nurse clinic

Possible diabetes (2)	9N1Q.00	Seen in diabetic clinic
Possible diabetes (2)	9N1i.00	Seen in diabetic foot clinic
Possible diabetes (2)	9N1o.00	Seen in multidisciplinary diabetic clinic
Possible diabetes (2)	9N1v.00	Seen in diabetic eye clinic
Possible diabetes (2)	9N2d.00	Seen by diabetologist
Possible diabetes (2)	9N2i.00	Seen by diabetic liaison nurse
Possible diabetes (2)	9N4I.00	DNA - Did not attend diabetic clinic
Possible diabetes (2)	9N4p.00	Did not attend diabetic retinopathy clinic
Possible diabetes (2)	9NM0.00	Attending diabetes clinic
Possible diabetes (2)	9NN8.00	Under care of diabetologist
Possible diabetes (2)	9NN9.00	Under care of diabetes specialist nurse
Possible diabetes (2)	9NND.00	Under care of diabetic foot screener
Possible diabetes (2)	9NiA.00	Did not attend diabetes structured education programme
Possible diabetes (2)	9NiD.00	Did not attend DESMOND diabetes structured education program
Possible diabetes (2)	9NiE.00	Did not attend XPERT diabetes structured education programme
Possible diabetes (2)	9Nl4.00	Seen by general practitioner special interest in diabetes
Possible diabetes (2)	9OL..00	Diabetes monitoring admin.
Possible diabetes (2)	9OL1.00	Attends diabetes monitoring
Possible diabetes (2)	9OL2.00	Refuses diabetes monitoring
Possible diabetes (2)	9OL3.00	Diabetes monitoring default
Possible diabetes (2)	9OL4.00	Diabetes monitoring 1st letter
Possible diabetes (2)	9OL5.00	Diabetes monitoring 2nd letter
Possible diabetes (2)	9OL6.00	Diabetes monitoring 3rd letter
Possible diabetes (2)	9OL7.00	Diabetes monitor.verbal invite
Possible diabetes (2)	9OL8.00	Diabetes monitor.phone invite
Possible diabetes (2)	9OLA.00	Diabetes monitor. check done
Possible diabetes (2)	9OLA.11	Diabetes monitored
Possible diabetes (2)	9OLB.00	Attended diabetes structured education programme
Possible diabetes (2)	9OLF.00	Diabetes structured education programme completed
Possible diabetes (2)	9OLG.00	Attended XPERT diabetes structured education programme
Possible diabetes (2)	9OLH.00	Attended DAFNE diabetes structured education programme
Possible diabetes (2)	9OLJ.00	DAFNE diabetes structured education programme completed
Possible diabetes (2)	9OLK.00	DESMOND diabetes structured education programme completed
Possible diabetes (2)	9OLL.00	XPERT diabetes structured education programme completed
Possible diabetes (2)	9OLM.00	Diabetes structured education programme declined
Possible diabetes (2)	9OLZ.00	Diabetes monitoring admin.NOS
Possible diabetes (2)	9h4..00	Exception reporting: diabetes quality indicators
Possible diabetes (2)	9h41.00	Excepted from diabetes qual indicators: Patient unsuitable
Possible diabetes (2)	9h42.00	Excepted from diabetes quality indicators: Informed dissent
Possible diabetes (2)	ZL22500	Under care of diabetic liaison nurse
Possible diabetes (2)	ZLA2500	Seen by diabetic liaison nurse
Possible diabetes (2)	ZLD7500	Discharge by diabetic liaison nurse

Possible diabetes (2)	ZRB4.00	Diabetes clinic satisfaction questionnaire
Possible diabetes (2)	ZRB4.11	CSQ - Diabetes clinic satisfaction questionnaire
Possible diabetes (2)	ZRB5.00	Diabetes treatment satisfaction questionnaire
Possible diabetes (2)	ZRB5.11	DTSQ - Diabetes treatment satisfaction questionnaire
Possible diabetes (2)	ZRB6.00	Diabetes wellbeing questionnaire
Possible diabetes (2)	ZRB6.11	DWBQ - Diabetes wellbeing questionnaire
Possible diabetes (2)	ZRBa.00	Education score - diabetes
Insulin dependent diabetes (3)	66An.00	Diabetes type 1 review
Insulin dependent diabetes (3)	C100000	Diabetes mellitus, juvenile type, no mention of complication
Insulin dependent diabetes (3)	C100011	Insulin dependent diabetes mellitus
Insulin dependent diabetes (3)	C101000	Diabetes mellitus, juvenile type, with ketoacidosis
Insulin dependent diabetes (3)	C102000	Diabetes mellitus, juvenile type, with hyperosmolar coma
Insulin dependent diabetes (3)	C103000	Diabetes mellitus, juvenile type, with ketoacidotic coma
Insulin dependent diabetes (3)	C104000	Diabetes mellitus, juvenile type, with renal manifestation
Insulin dependent diabetes (3)	C105000	Diabetes mellitus, juvenile type, + ophthalmic manifestation
Insulin dependent diabetes (3)	C106000	Diabetes mellitus, juvenile, + neurological manifestation
Insulin dependent diabetes (3)	C107000	Diabetes mellitus, juvenile +peripheral circulatory disorder
Insulin dependent diabetes (3)	C107300	IDDM with peripheral circulatory disorder
Insulin dependent diabetes (3)	C108.00	Insulin dependent diabetes mellitus
Insulin dependent diabetes (3)	C108.11	IDDM-Insulin dependent diabetes mellitus
Insulin dependent diabetes (3)	C108.12	Type 1 diabetes mellitus
Insulin dependent diabetes (3)	C108.13	Type I diabetes mellitus
Insulin dependent diabetes (3)	C108000	Insulin-dependent diabetes mellitus with renal complications
Insulin dependent diabetes (3)	C108011	Type I diabetes mellitus with renal complications
Insulin dependent diabetes (3)	C108012	Type 1 diabetes mellitus with renal complications
Insulin dependent diabetes (3)	C108100	Insulin-dependent diabetes mellitus with ophthalmic comps
Insulin dependent diabetes (3)	C108200	Insulin-dependent diabetes mellitus with neurological comps
Insulin dependent diabetes (3)	C108211	Type I diabetes mellitus with neurological complications
Insulin dependent diabetes (3)	C108212	Type 1 diabetes mellitus with neurological complications
Insulin dependent diabetes (3)	C108300	Insulin dependent diabetes mellitus with multiple complicatn
Insulin dependent diabetes (3)	C108400	Unstable insulin dependant diabetes mellitus
Insulin dependent diabetes (3)	C108411	Unstable type I diabetes mellitus
Insulin dependent diabetes (3)	C108412	Unstable type 1 diabetes mellitus
Insulin dependent diabetes (3)	C108500	Insulin dependent diabetes mellitus with ulcer
Insulin dependent diabetes (3)	C108511	Type I diabetes mellitus with ulcer
Insulin dependent diabetes (3)	C108512	Type 1 diabetes mellitus with ulcer
Insulin dependent diabetes (3)	C108600	Insulin dependent diabetes mellitus with gangrene
Insulin dependent diabetes (3)	C108700	Insulin dependent diabetes mellitus with retinopathy
Insulin dependent diabetes (3)	C108711	Type I diabetes mellitus with retinopathy
Insulin dependent diabetes (3)	C108712	Type 1 diabetes mellitus with retinopathy
Insulin dependent diabetes (3)	C108800	Insulin dependant diabetes mellitus - poor control
Insulin dependent diabetes (3)	C108811	Type I diabetes mellitus - poor control
Insulin dependent diabetes (3)	C108812	Type 1 diabetes mellitus - poor control

Insulin dependent diabetes (3)	C108900	Insulin dependant diabetes maturity onset
Insulin dependent diabetes (3)	C108911	Type I diabetes mellitus maturity onset
Insulin dependent diabetes (3)	C108912	Type 1 diabetes mellitus maturity onset
Insulin dependent diabetes (3)	C108A00	Insulin-dependent diabetes without complication
Insulin dependent diabetes (3)	C108A11	Type I diabetes mellitus without complication
Insulin dependent diabetes (3)	C108B00	Insulin dependent diabetes mellitus with mononeuropathy
Insulin dependent diabetes (3)	C108C00	Insulin dependent diabetes mellitus with polyneuropathy
Insulin dependent diabetes (3)	C108D00	Insulin dependent diabetes mellitus with nephropathy
Insulin dependent diabetes (3)	C108D11	Type I diabetes mellitus with nephropathy
Insulin dependent diabetes (3)	C108E00	Insulin dependent diabetes mellitus with hypoglycaemic coma
Insulin dependent diabetes (3)	C108E11	Type I diabetes mellitus with hypoglycaemic coma
Insulin dependent diabetes (3)	C108E12	Type 1 diabetes mellitus with hypoglycaemic coma
Insulin dependent diabetes (3)	C108F00	Insulin dependent diabetes mellitus with diabetic cataract
Insulin dependent diabetes (3)	C108F11	Type I diabetes mellitus with diabetic cataract
Insulin dependent diabetes (3)	C108G00	Insulin dependent diab mell with peripheral angiopathy
Insulin dependent diabetes (3)	C108H00	Insulin dependent diabetes mellitus with arthropathy
Insulin dependent diabetes (3)	C108H11	Type I diabetes mellitus with arthropathy
Insulin dependent diabetes (3)	C108J00	Insulin dependent diab mell with neuropathic arthropathy
Insulin dependent diabetes (3)	C108J11	Type I diabetes mellitus with neuropathic arthropathy
Insulin dependent diabetes (3)	C108J12	Type 1 diabetes mellitus with neuropathic arthropathy
Insulin dependent diabetes (3)	C10C.12	Maturity onset diabetes in youth type 1
Insulin dependent diabetes (3)	C10E.00	Type 1 diabetes mellitus
Insulin dependent diabetes (3)	C10E.11	Type I diabetes mellitus
Insulin dependent diabetes (3)	C10E.12	Insulin dependent diabetes mellitus
Insulin dependent diabetes (3)	C10E000	Type 1 diabetes mellitus with renal complications
Insulin dependent diabetes (3)	C10E100	Type 1 diabetes mellitus with ophthalmic complications
Insulin dependent diabetes (3)	C10E112	Insulin-dependent diabetes mellitus with ophthalmic comps
Insulin dependent diabetes (3)	C10E200	Type 1 diabetes mellitus with neurological complications
Insulin dependent diabetes (3)	C10E300	Type 1 diabetes mellitus with multiple complications
Insulin dependent diabetes (3)	C10E311	Type I diabetes mellitus with multiple complications
Insulin dependent diabetes (3)	C10E312	Insulin dependent diabetes mellitus with multiple complicat
Insulin dependent diabetes (3)	C10E400	Unstable type 1 diabetes mellitus
Insulin dependent diabetes (3)	C10E411	Unstable type I diabetes mellitus
Insulin dependent diabetes (3)	C10E412	Unstable insulin dependent diabetes mellitus
Insulin dependent diabetes (3)	C10E500	Type 1 diabetes mellitus with ulcer
Insulin dependent diabetes (3)	C10E511	Type I diabetes mellitus with ulcer
Insulin dependent diabetes (3)	C10E512	Insulin dependent diabetes mellitus with ulcer
Insulin dependent diabetes (3)	C10E600	Type 1 diabetes mellitus with gangrene
Insulin dependent diabetes (3)	C10E700	Type 1 diabetes mellitus with retinopathy
Insulin dependent diabetes (3)	C10E711	Type I diabetes mellitus with retinopathy
Insulin dependent diabetes (3)	C10E712	Insulin dependent diabetes mellitus with retinopathy
Insulin dependent diabetes (3)	C10E800	Type 1 diabetes mellitus - poor control
Insulin dependent diabetes (3)	C10E812	Insulin dependent diabetes mellitus - poor control
Insulin dependent diabetes (3)	C10E900	Type 1 diabetes mellitus maturity onset
Insulin dependent diabetes (3)	C10E911	Type I diabetes mellitus maturity onset

Insulin dependent diabetes (3)	C10E912	Insulin dependent diabetes maturity onset
Insulin dependent diabetes (3)	C10EA00	Type 1 diabetes mellitus without complication
Insulin dependent diabetes (3)	C10EA11	Type I diabetes mellitus without complication
Insulin dependent diabetes (3)	C10EB00	Type 1 diabetes mellitus with mononeuropathy
Insulin dependent diabetes (3)	C10EC00	Type 1 diabetes mellitus with polyneuropathy
Insulin dependent diabetes (3)	C10EC11	Type I diabetes mellitus with polyneuropathy
Insulin dependent diabetes (3)	C10ED00	Type 1 diabetes mellitus with nephropathy
Insulin dependent diabetes (3)	C10EE00	Type 1 diabetes mellitus with hypoglycaemic coma
Insulin dependent diabetes (3)	C10EF00	Type 1 diabetes mellitus with diabetic cataract
Insulin dependent diabetes (3)	C10EG00	Type 1 diabetes mellitus with peripheral angiopathy
Insulin dependent diabetes (3)	C10EH00	Type 1 diabetes mellitus with arthropathy
Insulin dependent diabetes (3)	C10EJ00	Type 1 diabetes mellitus with neuropathic arthropathy
Insulin dependent diabetes (3)	C10EK00	Type 1 diabetes mellitus with persistent proteinuria
Insulin dependent diabetes (3)	C10EL00	Type 1 diabetes mellitus with persistent microalbuminuria
Insulin dependent diabetes (3)	C10EM00	Type 1 diabetes mellitus with ketoacidosis
Insulin dependent diabetes (3)	C10EM11	Type I diabetes mellitus with ketoacidosis
Insulin dependent diabetes (3)	C10EN00	Type 1 diabetes mellitus with ketoacidotic coma
Insulin dependent diabetes (3)	C10EN11	Type I diabetes mellitus with ketoacidotic coma
Insulin dependent diabetes (3)	C10EP00	Type 1 diabetes mellitus with exudative maculopathy
Insulin dependent diabetes (3)	C10EP11	Type I diabetes mellitus with exudative maculopathy
Insulin dependent diabetes (3)	C10EQ00	Type 1 diabetes mellitus with gastroparesis
Insulin dependent diabetes (3)	C10z000	Diabetes mellitus, juvenile type, + unspecified complication
Insulin dependent diabetes (3)	L180500	Pre-existing diabetes mellitus, insulin-dependent
Insulin dependent diabetes (3)	M21yC00	Insulin lipohypertrophy
Insulin dependent diabetes (3)	M21yC11	Insulin site lipohypertrophy
Insulin dependent diabetes (3)	ZC2C900	Dietary advice for type I diabetes
Insulin dependent diabetes (3)	ZRbH.00	Perceived control of insulin-dependent diabetes
Non insulin dependent diabetes (4)	66Ao.00	Diabetes type 2 review
Non insulin dependent diabetes (4)	C100100	Diabetes mellitus, adult onset, no mention of complication
Non insulin dependent diabetes (4)	C100111	Maturity onset diabetes
Non insulin dependent diabetes (4)	C100112	Non-insulin dependent diabetes mellitus
Non insulin dependent diabetes (4)	C101100	Diabetes mellitus, adult onset, with ketoacidosis
Non insulin dependent diabetes (4)	C102100	Diabetes mellitus, adult onset, with hyperosmolar coma
Non insulin dependent diabetes (4)	C103100	Diabetes mellitus, adult onset, with ketoacidotic coma
Non insulin dependent diabetes (4)	C104100	Diabetes mellitus, adult onset, with renal manifestation
Non insulin dependent diabetes (4)	C105100	Diabetes mellitus, adult onset, + ophthalmic manifestation
Non insulin dependent diabetes (4)	C106100	Diabetes mellitus, adult onset, + neurological manifestation
Non insulin dependent diabetes (4)	C107100	Diabetes mellitus, adult, + peripheral circulatory disorder
Non insulin dependent diabetes (4)	C107200	Diabetes mellitus, adult with gangrene
Non insulin dependent diabetes (4)	C107400	NIDDM with peripheral circulatory disorder
Non insulin dependent diabetes (4)	C109.00	Non-insulin dependent diabetes mellitus
Non insulin dependent diabetes (4)	C109.11	NIDDM - Non-insulin dependent diabetes mellitus
Non insulin dependent diabetes (4)	C109.12	Type 2 diabetes mellitus
Non insulin dependent diabetes (4)	C109.13	Type II diabetes mellitus

Non insulin dependent diabetes (4)	C109000	Non-insulin-dependent diabetes mellitus with renal comps
Non insulin dependent diabetes (4)	C109011	Type II diabetes mellitus with renal complications
Non insulin dependent diabetes (4)	C109012	Type 2 diabetes mellitus with renal complications
Non insulin dependent diabetes (4)	C109100	Non-insulin-dependent diabetes mellitus with ophthalm comps
Non insulin dependent diabetes (4)	C109111	Type II diabetes mellitus with ophthalmic complications
Non insulin dependent diabetes (4)	C109112	Type 2 diabetes mellitus with ophthalmic complications
Non insulin dependent diabetes (4)	C109200	Non-insulin-dependent diabetes mellitus with neuro comps
Non insulin dependent diabetes (4)	C109211	Type II diabetes mellitus with neurological complications
Non insulin dependent diabetes (4)	C109212	Type 2 diabetes mellitus with neurological complications
Non insulin dependent diabetes (4)	C109300	Non-insulin-dependent diabetes mellitus with multiple comps
Non insulin dependent diabetes (4)	C109400	Non-insulin dependent diabetes mellitus with ulcer
Non insulin dependent diabetes (4)	C109411	Type II diabetes mellitus with ulcer
Non insulin dependent diabetes (4)	C109412	Type 2 diabetes mellitus with ulcer
Non insulin dependent diabetes (4)	C109500	Non-insulin dependent diabetes mellitus with gangrene
Non insulin dependent diabetes (4)	C109511	Type II diabetes mellitus with gangrene
Non insulin dependent diabetes (4)	C109512	Type 2 diabetes mellitus with gangrene
Non insulin dependent diabetes (4)	C109600	Non-insulin-dependent diabetes mellitus with retinopathy
Non insulin dependent diabetes (4)	C109611	Type II diabetes mellitus with retinopathy
Non insulin dependent diabetes (4)	C109612	Type 2 diabetes mellitus with retinopathy
Non insulin dependent diabetes (4)	C109700	Non-insulin dependant diabetes mellitus - poor control
Non insulin dependent diabetes (4)	C109711	Type II diabetes mellitus - poor control
Non insulin dependent diabetes (4)	C109712	Type 2 diabetes mellitus - poor control
Non insulin dependent diabetes (4)	C109900	Non-insulin-dependent diabetes mellitus without complication
Non insulin dependent diabetes (4)	C109A00	Non-insulin dependent diabetes mellitus with mononeuropathy
Non insulin dependent diabetes (4)	C109A11	Type II diabetes mellitus with mononeuropathy
Non insulin dependent diabetes (4)	C109B00	Non-insulin dependent diabetes mellitus with polyneuropathy
Non insulin dependent diabetes (4)	C109B11	Type II diabetes mellitus with polyneuropathy
Non insulin dependent diabetes (4)	C109C00	Non-insulin dependent diabetes mellitus with nephropathy
Non insulin dependent diabetes (4)	C109C11	Type II diabetes mellitus with nephropathy
Non insulin dependent diabetes (4)	C109C12	Type 2 diabetes mellitus with nephropathy
Non insulin dependent diabetes (4)	C109D00	Non-insulin dependent diabetes mellitus with hypoglyca coma
Non insulin dependent diabetes (4)	C109D11	Type II diabetes mellitus with hypoglycaemic coma
Non insulin dependent diabetes (4)	C109D12	Type 2 diabetes mellitus with hypoglycaemic coma
Non insulin dependent diabetes (4)	C109E00	Non-insulin depend diabetes mellitus with diabetic cataract
Non insulin dependent diabetes (4)	C109E11	Type II diabetes mellitus with diabetic cataract
Non insulin dependent diabetes (4)	C109E12	Type 2 diabetes mellitus with diabetic cataract
Non insulin dependent diabetes (4)	C109F00	Non-insulin-dependent d m with peripheral angiopath
Non insulin dependent diabetes (4)	C109F11	Type II diabetes mellitus with peripheral angiopathy
Non insulin dependent diabetes (4)	C109F12	Type 2 diabetes mellitus with peripheral angiopathy
Non insulin dependent diabetes (4)	C109G00	Non-insulin dependent diabetes mellitus with arthropathy

Non insulin dependent diabetes (4)	C109G11	Type II diabetes mellitus with arthropathy
Non insulin dependent diabetes (4)	C109G12	Type 2 diabetes mellitus with arthropathy
Non insulin dependent diabetes (4)	C109H00	Non-insulin dependent d m with neuropathic arthropathy
Non insulin dependent diabetes (4)	C109H11	Type II diabetes mellitus with neuropathic arthropathy
Non insulin dependent diabetes (4)	C109H12	Type 2 diabetes mellitus with neuropathic arthropathy
Non insulin dependent diabetes (4)	C109J00	Insulin treated Type 2 diabetes mellitus
Non insulin dependent diabetes (4)	C109J11	Insulin treated non-insulin dependent diabetes mellitus
Non insulin dependent diabetes (4)	C109J12	Insulin treated Type II diabetes mellitus
Non insulin dependent diabetes (4)	C109K00	Hyperosmolar non-ketotic state in type 2 diabetes mellitus
Non insulin dependent diabetes (4)	C10C.11	Maturity onset diabetes in youth
Non insulin dependent diabetes (4)	C10D.00	Diabetes mellitus autosomal dominant type 2
Non insulin dependent diabetes (4)	C10D.11	Maturity onset diabetes in youth type 2
Non insulin dependent diabetes (4)	C10ER00	Latent autoimmune diabetes mellitus in adult
Non insulin dependent diabetes (4)	C10F.00	Type 2 diabetes mellitus
Non insulin dependent diabetes (4)	C10F.11	Type II diabetes mellitus
Non insulin dependent diabetes (4)	C10F000	Type 2 diabetes mellitus with renal complications
Non insulin dependent diabetes (4)	C10F011	Type II diabetes mellitus with renal complications
Non insulin dependent diabetes (4)	C10F100	Type 2 diabetes mellitus with ophthalmic complications
Non insulin dependent diabetes (4)	C10F200	Type 2 diabetes mellitus with neurological complications
Non insulin dependent diabetes (4)	C10F211	Type II diabetes mellitus with neurological complications
Non insulin dependent diabetes (4)	C10F300	Type 2 diabetes mellitus with multiple complications
Non insulin dependent diabetes (4)	C10F311	Type II diabetes mellitus with multiple complications
Non insulin dependent diabetes (4)	C10F400	Type 2 diabetes mellitus with ulcer
Non insulin dependent diabetes (4)	C10F411	Type II diabetes mellitus with ulcer
Non insulin dependent diabetes (4)	C10F500	Type 2 diabetes mellitus with gangrene
Non insulin dependent diabetes (4)	C10F600	Type 2 diabetes mellitus with retinopathy
Non insulin dependent diabetes (4)	C10F611	Type II diabetes mellitus with retinopathy
Non insulin dependent diabetes (4)	C10F700	Type 2 diabetes mellitus - poor control
Non insulin dependent diabetes (4)	C10F711	Type II diabetes mellitus - poor control
Non insulin dependent diabetes (4)	C10F900	Type 2 diabetes mellitus without complication
Non insulin dependent diabetes (4)	C10F911	Type II diabetes mellitus without complication
Non insulin dependent diabetes (4)	C10FA00	Type 2 diabetes mellitus with mononeuropathy
Non insulin dependent diabetes (4)	C10FA11	Type II diabetes mellitus with mononeuropathy
Non insulin dependent diabetes (4)	C10FB00	Type 2 diabetes mellitus with polyneuropathy
Non insulin dependent diabetes (4)	C10FB11	Type II diabetes mellitus with polyneuropathy
Non insulin dependent diabetes (4)	C10FC00	Type 2 diabetes mellitus with nephropathy
Non insulin dependent diabetes (4)	C10FD00	Type 2 diabetes mellitus with hypoglycaemic coma
Non insulin dependent diabetes (4)	C10FD11	Type II diabetes mellitus with hypoglycaemic coma
Non insulin dependent diabetes (4)	C10FE00	Type 2 diabetes mellitus with diabetic cataract
Non insulin dependent diabetes (4)	C10FE11	Type II diabetes mellitus with diabetic cataract
Non insulin dependent diabetes (4)	C10FF00	Type 2 diabetes mellitus with peripheral angiopathy
Non insulin dependent diabetes (4)	C10FG00	Type 2 diabetes mellitus with arthropathy
Non insulin dependent diabetes (4)	C10FH00	Type 2 diabetes mellitus with neuropathic arthropathy
Non insulin dependent diabetes (4)	C10FJ00	Insulin treated Type 2 diabetes mellitus
Non insulin dependent diabetes (4)	C10FJ11	Insulin treated Type II diabetes mellitus
Non insulin dependent diabetes (4)	C10FK00	Hyperosmolar non-ketotic state in type 2 diabetes mellitus

Non insulin dependent diabetes (4)	C10FL00	Type 2 diabetes mellitus with persistent proteinuria
Non insulin dependent diabetes (4)	C10FL11	Type II diabetes mellitus with persistent proteinuria
Non insulin dependent diabetes (4)	C10FM00	Type 2 diabetes mellitus with persistent microalbuminuria
Non insulin dependent diabetes (4)	C10FM11	Type II diabetes mellitus with persistent microalbuminuria
Non insulin dependent diabetes (4)	C10FN00	Type 2 diabetes mellitus with ketoacidosis
Non insulin dependent diabetes (4)	C10FP00	Type 2 diabetes mellitus with ketoacidotic coma
Non insulin dependent diabetes (4)	C10FQ00	Type 2 diabetes mellitus with exudative maculopathy
Non insulin dependent diabetes (4)	C10FR00	Type 2 diabetes mellitus with gastroparesis
Non insulin dependent diabetes (4)	C10y100	Diabetes mellitus, adult, + other specified manifestation
Non insulin dependent diabetes (4)	C10z100	Diabetes mellitus, adult onset, + unspecified complication
Non insulin dependent diabetes (4)	L180600	Pre-existing diabetes mellitus, non-insulin-dependent
Non insulin dependent diabetes (4)	ZC2CA00	Dietary advice for type II diabetes
Secondary diabetes (5)	C10B.00	Diabetes mellitus induced by steroids
Secondary diabetes (5)	C10B000	Steroid induced diabetes mellitus without complication
Secondary diabetes (5)	C10FS00	Maternally inherited diabetes mellitus
Secondary diabetes (5)	C10G.00	Secondary pancreatic diabetes mellitus
Secondary diabetes (5)	C10G000	Secondary pancreatic diabetes mellitus without complication
Secondary diabetes (5)	C10H.00	Diabetes mellitus induced by non-steroid drugs
Secondary diabetes (5)	C10H000	DM induced by non-steroid drugs without complication
Secondary diabetes (5)	C10N.00	Secondary diabetes mellitus
Secondary diabetes (5)	C10N000	Secondary diabetes mellitus without complication
Secondary diabetes (5)	C10N100	Cystic fibrosis related diabetes mellitus
Secondary diabetes (5)	C11y000	Steroid induced diabetes
Diabetes, not otherwise specified (6)	13AB.00	Diabetic lipid lowering diet
Diabetes, not otherwise specified (6)	13AC.00	Diabetic weight reducing diet
Diabetes, not otherwise specified (6)	13B1.00	Diabetic diet
Diabetes, not otherwise specified (6)	2BBF.00	Retinal abnormality - diabetes related
Diabetes, not otherwise specified (6)	2BBL.00	O/E - diabetic maculopathy present both eyes
Diabetes, not otherwise specified (6)	2BBM.00	O/E - diabetic maculopathy absent both eyes
Diabetes, not otherwise specified (6)	2BBP.00	O/E - right eye background diabetic retinopathy
Diabetes, not otherwise specified (6)	2BBQ.00	O/E - left eye background diabetic retinopathy
Diabetes, not otherwise specified (6)	2BBR.00	O/E - right eye preproliferative diabetic retinopathy
Diabetes, not otherwise specified (6)	2BBS.00	O/E - left eye preproliferative diabetic retinopathy
Diabetes, not otherwise specified (6)	2BBT.00	O/E - right eye proliferative diabetic retinopathy
Diabetes, not otherwise specified (6)	2BBV.00	O/E - left eye proliferative diabetic retinopathy
Diabetes, not otherwise specified (6)	2BBW.00	O/E - right eye diabetic maculopathy
Diabetes, not otherwise specified (6)	2BBX.00	O/E - left eye diabetic maculopathy
Diabetes, not otherwise specified (6)	2BBk.00	O/E - right eye stable treated prolif diabetic retinopathy
Diabetes, not otherwise specified (6)	2BBl.00	O/E - left eye stable treated prolif diabetic retinopathy
Diabetes, not otherwise specified (6)	2BBo.00	O/E - sight threatening diabetic retinopathy
Diabetes, not otherwise specified (6)	2G51000	Foot abnormality - diabetes related
Diabetes, not otherwise specified (6)	2G5A.00	O/E - Right diabetic foot at risk
Diabetes, not otherwise specified (6)	2G5B.00	O/E - Left diabetic foot at risk
Diabetes, not otherwise specified (6)	2G5C.00	Foot abnormality - diabetes related
Diabetes, not otherwise specified (6)	2G5E.00	O/E - Right diabetic foot at low risk

Diabetes, not otherwise specified (6)	2G5F.00	O/E - Right diabetic foot at moderate risk
Diabetes, not otherwise specified (6)	2G5G.00	O/E - Right diabetic foot at high risk
Diabetes, not otherwise specified (6)	2G5H.00	O/E - Right diabetic foot - ulcerated
Diabetes, not otherwise specified (6)	2G5I.00	O/E - Left diabetic foot at low risk
Diabetes, not otherwise specified (6)	2G5J.00	O/E - Left diabetic foot at moderate risk
Diabetes, not otherwise specified (6)	2G5K.00	O/E - Left diabetic foot at high risk
Diabetes, not otherwise specified (6)	2G5L.00	O/E - Left diabetic foot - ulcerated
Diabetes, not otherwise specified (6)	2G5V.00	O/E - right chronic diabetic foot ulcer
Diabetes, not otherwise specified (6)	2G5W.00	O/E - left chronic diabetic foot ulcer
Diabetes, not otherwise specified (6)	66A3.00	Diabetic on diet only
Diabetes, not otherwise specified (6)	66A4.00	Diabetic on oral treatment
Diabetes, not otherwise specified (6)	66A5.00	Diabetic on insulin
Diabetes, not otherwise specified (6)	66A8.00	Has seen dietician - diabetes
Diabetes, not otherwise specified (6)	66A9.00	Understands diet - diabetes
Diabetes, not otherwise specified (6)	66AA.11	Injection sites - diabetic
Diabetes, not otherwise specified (6)	66AD.00	Fundoscopy - diabetic check
Diabetes, not otherwise specified (6)	66AG.00	Diabetic drug side effects
Diabetes, not otherwise specified (6)	66AH.00	Diabetic treatment changed
Diabetes, not otherwise specified (6)	66AI.00	Diabetic - good control
Diabetes, not otherwise specified (6)	66AJ.00	Diabetic - poor control
Diabetes, not otherwise specified (6)	66AJ.11	Unstable diabetes
Diabetes, not otherwise specified (6)	66AJ100	Brittle diabetes
Diabetes, not otherwise specified (6)	66AJz00	Diabetic - poor control NOS
Diabetes, not otherwise specified (6)	66AK.00	Diabetic - cooperative patient
Diabetes, not otherwise specified (6)	66AL.00	Diabetic-uncooperative patient
Diabetes, not otherwise specified (6)	66AN.00	Date diabetic treatment start
Diabetes, not otherwise specified (6)	66AO.00	Date diabetic treatment stopp.
Diabetes, not otherwise specified (6)	66AP.00	Diabetes: practice programme
Diabetes, not otherwise specified (6)	66AQ.00	Diabetes: shared care programme
Diabetes, not otherwise specified (6)	66AR.00	Diabetes management plan given
Diabetes, not otherwise specified (6)	66AS.00	Diabetic annual review
Diabetes, not otherwise specified (6)	66AT.00	Annual diabetic blood test
Diabetes, not otherwise specified (6)	66AU.00	Diabetes care by hospital only
Diabetes, not otherwise specified (6)	66AV.00	Diabetic on insulin and oral treatment
Diabetes, not otherwise specified (6)	66AW.00	Diabetic foot risk assessment
Diabetes, not otherwise specified (6)	66AX.00	Diabetes: shared care in pregnancy - diabetol and obstet
Diabetes, not otherwise specified (6)	66AY.00	Diabetic diet - good compliance
Diabetes, not otherwise specified (6)	66Aa.00	Diabetic diet - poor compliance
Diabetes, not otherwise specified (6)	66Ab.00	Diabetic foot examination
Diabetes, not otherwise specified (6)	66Ac.00	Diabetic peripheral neuropathy screening
Diabetes, not otherwise specified (6)	66Ag.00	Insulin needles changed daily
Diabetes, not otherwise specified (6)	66Ah.00	Insulin needles changed for each injection
Diabetes, not otherwise specified (6)	66Ai.00	Diabetic 6 month review
Diabetes, not otherwise specified (6)	66Aj.00	Insulin needles changed less than once a day
Diabetes, not otherwise specified (6)	66Am.00	Insulin dose changed
Diabetes, not otherwise specified (6)	66Ap.00	Insulin treatment initiated
Diabetes, not otherwise specified (6)	66Aq.00	Diabetic foot screen
Diabetes, not otherwise specified (6)	6761	Diabetic pre-pregnancy counselling

Diabetes, not otherwise specified (6)	68A7.00	Diabetic retinopathy screening
Diabetes, not otherwise specified (6)	68A9.00	Diabetic retinopathy screening offered
Diabetes, not otherwise specified (6)	68AB.00	Diabetic digital retinopathy screening offered
Diabetes, not otherwise specified (6)	7276	Pan retinal photocoagulation for diabetes
Diabetes, not otherwise specified (6)	7L10000	Continuous subcutaneous infusion of insulin
Diabetes, not otherwise specified (6)	7L19800	Subcutaneous injection of insulin
Diabetes, not otherwise specified (6)	889A.00	Diab mellit insulin-glucose infus acute myocardial infarct
Diabetes, not otherwise specified (6)	8A13.00	Diabetic stabilisation
Diabetes, not otherwise specified (6)	8B3l.00	Diabetes medication review
Diabetes, not otherwise specified (6)	8BL2.00	Patient on maximal tolerated therapy for diabetes
Diabetes, not otherwise specified (6)	8CA4100	Pt advised re diabetic diet
Diabetes, not otherwise specified (6)	8CAQ.00	Advice about blood glucose control
Diabetes, not otherwise specified (6)	8CP2.00	Transition of diabetes care options discussed
Diabetes, not otherwise specified (6)	8H2J.00	Admit diabetic emergency
Diabetes, not otherwise specified (6)	8H3O.00	Non-urgent diabetic admission
Diabetes, not otherwise specified (6)	8H7r.00	Refer to diabetic foot screener
Diabetes, not otherwise specified (6)	8HBG.00	Diabetic retinopathy 12 month review
Diabetes, not otherwise specified (6)	8HBH.00	Diabetic retinopathy 6 month review
Diabetes, not otherwise specified (6)	8HLE.00	Diabetology D.V. done
Diabetes, not otherwise specified (6)	8Hl1.00	Referral for diabetic retinopathy screening
Diabetes, not otherwise specified (6)	8I3W.00	Diabetic foot examination declined
Diabetes, not otherwise specified (6)	8I3X.00	Diabetic retinopathy screening refused
Diabetes, not otherwise specified (6)	8I3k.00	Insulin therapy declined
Diabetes, not otherwise specified (6)	8I57.00	Patient held diabetic record declined
Diabetes, not otherwise specified (6)	9360	Patient held diabetic record issued
Diabetes, not otherwise specified (6)	9OLD.00	Diabetic patient unsuitable for digital retinal photography
Diabetes, not otherwise specified (6)	C10..00	Diabetes mellitus
Diabetes, not otherwise specified (6)	C100.00	Diabetes mellitus with no mention of complication
Diabetes, not otherwise specified (6)	C100z00	Diabetes mellitus NOS with no mention of complication
Diabetes, not otherwise specified (6)	C101.00	Diabetes mellitus with ketoacidosis
Diabetes, not otherwise specified (6)	C101y00	Other specified diabetes mellitus with ketoacidosis
Diabetes, not otherwise specified (6)	C101z00	Diabetes mellitus NOS with ketoacidosis
Diabetes, not otherwise specified (6)	C102.00	Diabetes mellitus with hyperosmolar coma
Diabetes, not otherwise specified (6)	C102z00	Diabetes mellitus NOS with hyperosmolar coma
Diabetes, not otherwise specified (6)	C103.00	Diabetes mellitus with ketoacidotic coma
Diabetes, not otherwise specified (6)	C103y00	Other specified diabetes mellitus with coma
Diabetes, not otherwise specified (6)	C103z00	Diabetes mellitus NOS with ketoacidotic coma
Diabetes, not otherwise specified (6)	C104.00	Diabetes mellitus with renal manifestation
Diabetes, not otherwise specified (6)	C104.11	Diabetic nephropathy
Diabetes, not otherwise specified (6)	C104y00	Other specified diabetes mellitus with renal complications
Diabetes, not otherwise specified (6)	C104z00	Diabetes mellitis with nephropathy NOS
Diabetes, not otherwise specified (6)	C105.00	Diabetes mellitus with ophthalmic manifestation
Diabetes, not otherwise specified (6)	C105y00	Other specified diabetes mellitus with ophthalmic complicatn
Diabetes, not otherwise specified (6)	C105z00	Diabetes mellitus NOS with ophthalmic manifestation
Diabetes, not otherwise specified (6)	C106.00	Diabetes mellitus with neurological manifestation

Diabetes, not otherwise specified (6)	C106.11	Diabetic amyotrophy
Diabetes, not otherwise specified (6)	C106.12	Diabetes mellitus with neuropathy
Diabetes, not otherwise specified (6)	C106.13	Diabetes mellitus with polyneuropathy
Diabetes, not otherwise specified (6)	C106y00	Other specified diabetes mellitus with neurological comps
Diabetes, not otherwise specified (6)	C106z00	Diabetes mellitus NOS with neurological manifestation
Diabetes, not otherwise specified (6)	C107.00	Diabetes mellitus with peripheral circulatory disorder
Diabetes, not otherwise specified (6)	C107.11	Diabetes mellitus with gangrene
Diabetes, not otherwise specified (6)	C107.12	Diabetes with gangrene
Diabetes, not otherwise specified (6)	C107z00	Diabetes mellitus NOS with peripheral circulatory disorder
Diabetes, not otherwise specified (6)	C108y00	Other specified diabetes mellitus with multiple comps
Diabetes, not otherwise specified (6)	C108z00	Unspecified diabetes mellitus with multiple complications
Diabetes, not otherwise specified (6)	C10A.00	Malnutrition-related diabetes mellitus
Diabetes, not otherwise specified (6)	C10A000	Malnutrition-related diabetes mellitus with coma
Diabetes, not otherwise specified (6)	C10A100	Malnutrition-related diabetes mellitus with ketoacidosis
Diabetes, not otherwise specified (6)	C10C.00	Diabetes mellitus autosomal dominant
Diabetes, not otherwise specified (6)	C10M.00	Lipoatrophic diabetes mellitus
Diabetes, not otherwise specified (6)	C10y.00	Diabetes mellitus with other specified manifestation
Diabetes, not otherwise specified (6)	C10yy00	Other specified diabetes mellitus with other spec comps
Diabetes, not otherwise specified (6)	C10yz00	Diabetes mellitus NOS with other specified manifestation
Diabetes, not otherwise specified (6)	C10z.00	Diabetes mellitus with unspecified complication
Diabetes, not otherwise specified (6)	C10zy00	Other specified diabetes mellitus with unspecified comps
Diabetes, not otherwise specified (6)	C10zz00	Diabetes mellitus NOS with unspecified complication
Diabetes, not otherwise specified (6)	C314.11	Renal diabetes
Diabetes, not otherwise specified (6)	C350011	Bronzed diabetes
Diabetes, not otherwise specified (6)	Cyu2.00	[X]Diabetes mellitus
Diabetes, not otherwise specified (6)	Cyu2000	[X]Other specified diabetes mellitus
Diabetes, not otherwise specified (6)	F171100	Autonomic neuropathy due to diabetes
Diabetes, not otherwise specified (6)	F345000	Diabetic mononeuritis multiplex
Diabetes, not otherwise specified (6)	F35z000	Diabetic mononeuritis NOS
Diabetes, not otherwise specified (6)	F372.00	Polyneuropathy in diabetes
Diabetes, not otherwise specified (6)	F372.11	Diabetic polyneuropathy
Diabetes, not otherwise specified (6)	F372.12	Diabetic neuropathy
Diabetes, not otherwise specified (6)	F372000	Acute painful diabetic neuropathy
Diabetes, not otherwise specified (6)	F372100	Chronic painful diabetic neuropathy
Diabetes, not otherwise specified (6)	F372200	Asymptomatic diabetic neuropathy
Diabetes, not otherwise specified (6)	F381300	Myasthenic syndrome due to diabetic amyotrophy
Diabetes, not otherwise specified (6)	F381311	Diabetic amyotrophy
Diabetes, not otherwise specified (6)	F3y0.00	Diabetic mononeuropathy
Diabetes, not otherwise specified (6)	F420.00	Diabetic retinopathy
Diabetes, not otherwise specified (6)	F420000	Background diabetic retinopathy
Diabetes, not otherwise specified (6)	F420100	Proliferative diabetic retinopathy
Diabetes, not otherwise specified (6)	F420200	Preproliferative diabetic retinopathy
Diabetes, not otherwise specified (6)	F420300	Advanced diabetic maculopathy
Diabetes, not otherwise specified (6)	F420400	Diabetic maculopathy
Diabetes, not otherwise specified (6)	F420500	Advanced diabetic retinal disease

Diabetes, not otherwise specified (6)	F420600	Non proliferative diabetic retinopathy
Diabetes, not otherwise specified (6)	F420700	High risk proliferative diabetic retinopathy
Diabetes, not otherwise specified (6)	F420800	High risk non proliferative diabetic retinopathy
Diabetes, not otherwise specified (6)	F420z00	Diabetic retinopathy NOS
Diabetes, not otherwise specified (6)	F440700	Diabetic iritis
Diabetes, not otherwise specified (6)	F464000	Diabetic cataract
Diabetes, not otherwise specified (6)	G73y000	Diabetic peripheral angiopathy
Diabetes, not otherwise specified (6)	K01x100	Nephrotic syndrome in diabetes mellitus
Diabetes, not otherwise specified (6)	K01x111	Kimmelstiel - Wilson disease
Diabetes, not otherwise specified (6)	L180X00	Pre-existing diabetes mellitus, unspecified
Diabetes, not otherwise specified (6)	M037200	Cellulitis in diabetic foot
Diabetes, not otherwise specified (6)	M271000	Ischaemic ulcer diabetic foot
Diabetes, not otherwise specified (6)	M271100	Neuropathic diabetic ulcer - foot
Diabetes, not otherwise specified (6)	M271200	Mixed diabetic ulcer - foot
Diabetes, not otherwise specified (6)	N030000	Diabetic cheiroarthropathy
Diabetes, not otherwise specified (6)	N030011	Diabetic cheiropathy
Diabetes, not otherwise specified (6)	N030100	Diabetic Charcot arthropathy
Diabetes, not otherwise specified (6)	Q441.00	Neonatal diabetes mellitus
Diabetes, not otherwise specified (6)	R054200	[D]Gangrene of toe in diabetic
Diabetes, not otherwise specified (6)	R054300	[D]Widespread diabetic foot gangrene
Diabetes, not otherwise specified (6)	TJ23.00	Adverse reaction to insulins and antidiabetic agents
Diabetes, not otherwise specified (6)	TJ23z00	Adverse reaction to insulins and antidiabetic agents NOS
Diabetes, not otherwise specified (6)	U602311	[X] Adverse reaction to insulins and antidiabetic agents
Diabetes, not otherwise specified (6)	ZC2C800	Dietary advice for diabetes mellitus
Diabetes, not otherwise specified (6)	ZV65312	[V]Dietary counselling in diabetes mellitus
Diabetes excluded (7)	1I0..00	Diabetes mellitus excluded

Table S2. ICD-10 code list from secondary care EHR data source

Category (code)	ICD10 code	ICD10 term
Insulin dependent diabetes (3)	E10	Insulin-dependent diabetes mellitus
Non insulin dependent diabetes (4)	E11	Non-insulin-dependent diabetes mellitus
Secondary diabetes (5)	E12	Malnutrition-related diabetes mellitus
Secondary diabetes (5)	O242	Diabetes mellitus in pregnancy: Pre-existing malnutrition-related diabetes mellitus
Diabetes not otherwise specified (6)	E13	Other specified diabetes mellitus
Diabetes not otherwise specified (6)	E14	Unspecified diabetes mellitus
Diabetes not otherwise specified (6)	G590	Diabetic mononeuropathy
Diabetes not otherwise specified (6)	G632	Diabetic polyneuropathy
Diabetes not otherwise specified (6)	H280	Diabetic cataract
Diabetes not otherwise specified (6)	H360	Diabetic retinopathy
Diabetes not otherwise specified (6)	M142	Diabetic arthropathy
Diabetes not otherwise specified (6)	N083	Glomerular disorders in diabetes mellitus
Diabetes not otherwise specified (6)	O240	Diabetes mellitus in pregnancy: Pre-existing diabetes mellitus, insulin-dependent
Diabetes not otherwise specified (6)	O241	Diabetes mellitus in pregnancy: Pre-existing diabetes mellitus, non-insulin-dependent
Diabetes not otherwise specified (6)	O243	Diabetes mellitus in pregnancy: Pre-existing diabetes mellitus, unspecified

Figure S1. Diabetes flowchart representation

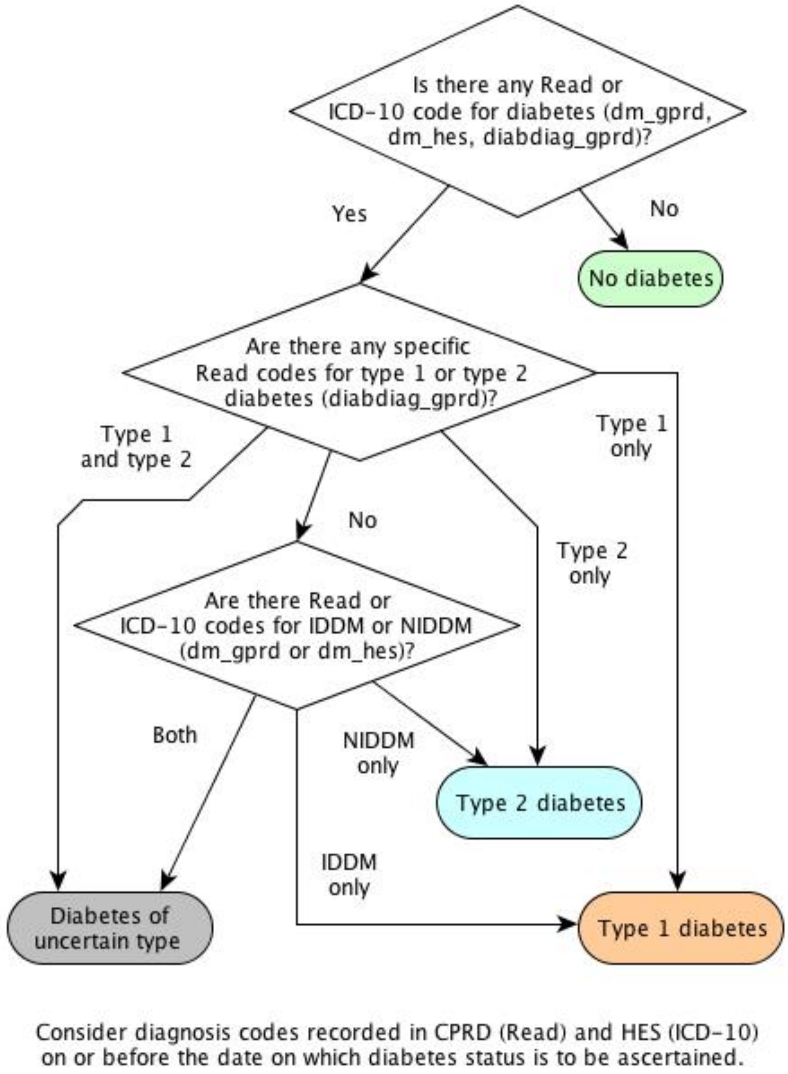

Calculating ethnic-specific BMI cut-off points

As our models included fractional polynomials, the back calculations were performed using numerical rather than analytical approach. Consider the log incidence rate (ir) of T2DM for the fitted model which takes the form:

$$\log(ir) = \beta_0 + \beta_1 bmi^{p1} + \beta_2 bmi^{p2} + \beta_{31} eth_1 + \beta_{32} eth_2 + ...$$

where bmi is body mass index, ethi is dummy variable for ethnicity group i, β's are the respective estimated coefficients and p1 and p2 are estimated powers for bmi.

After estimating all coefficients and powers for the fractional polynomials as well as predicted ir at bmi=30 for the white ethnic group (all done using stata), we can rearrange the above equation as follows:

$$\beta_1 bmi^{p1} + \beta_2 bmi^{p2} + (\beta_0 + \beta_{31} eth_1 + \beta_{32} eth_2 + ...) - \log(ir) = 0$$
$$\beta_1 bmi^{p1} + \beta_2 bmi^{p2} + \underbrace{(\beta_0 + \beta_{31} eth_1 + \beta_{32} eth_2 + ...) - \log(ir)}_{C} = 0$$
$$\beta_1 bmi^{p1} + \beta_2 bmi^{p2} + C = 0 \ldots *$$

And then calculate the bmi cut-off for each the remaining ethnicity groups by looking for a bmi value that solves the equation (*) above to zero.

Table S3. Ethnic breakdown of study population

Ethnic group	n	%
White	1,333,816	90.56
Black African	35,342	2.40
Black Caribbean	4,394	0.30
Black British	5,250	0.36
Black Other	4,168	0.28
Bangladeshi	5,316	0.36
Indian	48,353	3.28
Nepali	937	0.06
Pakistani	18,565	1.26
Sri Lankan	2,269	0.15
Tamil	0.15	0.01
Chinese	10,934	0.74
Arab	2,764	0.19
Missing	587	0.04
Total	1,472,819	100.00

Figure S2. Type 2 diabetes mellitus incidence rates during median 6.5 years follow-up by baseline body mass index among White, black, South Asian, Chinese and Arab populations in England. Negative binomial regression model adjusted for age, sex, smoking status and social deprivation.

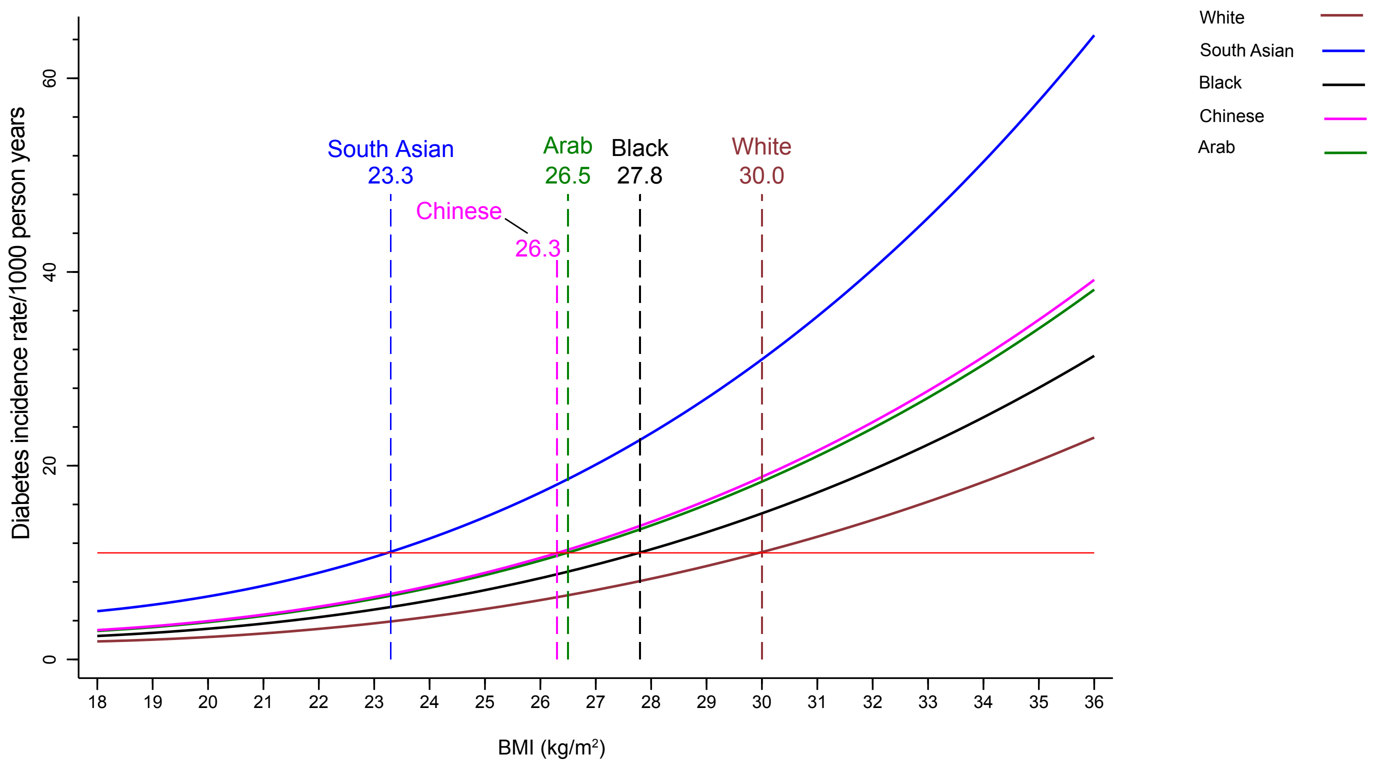

Supplement: Supplementary appendix [file mmc1.pdf]
